# Barcoding Atlantic Canada’s mesopelagic and upper bathypelagic marine fishes

**DOI:** 10.1371/journal.pone.0185173

**Published:** 2017-09-20

**Authors:** Ellen L. Kenchington, Shauna M. Baillie, Trevor J. Kenchington, Paul Bentzen

**Affiliations:** 1 Department of Fisheries and Oceans, Bedford Institute of Oceanography, Dartmouth, Nova Scotia, Canada; 2 Department of Biology, Dalhousie University, Halifax, Nova Scotia, Canada; Chang Gung University, TAIWAN

## Abstract

DNA barcode sequences were developed from 557 mesopelagic and upper bathypelagic teleost specimens collected in waters off Atlantic Canada. Confident morphological identifications were available for 366 specimens, of 118 species and 93 genera, which yielded 328 haplotypes. Five of the species were novel to the Barcode of Life Database (BOLD). Most of the 118 species conformed to expectations of monophyly and the presence of a “barcode gap”, though some known weaknesses in existing taxonomy were confirmed and a deficiency in published keys was revealed. Of the specimens for which no firm morphological identification was available, 156 were successfully identified to species, and a further 11 to genus, using their barcode sequences and a combination of distance- and character-based methods. The remaining 24 specimens were from species for which no reference barcode is yet available or else ones confused by apparent misidentification of publicly available sequences in BOLD. Addition of the new sequences to those previously in BOLD contributed support to recent taxonomic revisions of *Chiasmodon* and *Poromitra*, while it also revealed 18 cases of potential cryptic speciation. Most of the latter appear to result from genetic divergence among populations in different ocean basins, while the general lack of strong horizontal environmental gradients within the deep sea has allowed morphology to be conserved. Other examples of divergence appear to distinguish individuals living under the sub-tropical gyre of the North Atlantic from those under that ocean’s sub-polar gyre. In contrast, the available sequences for two myctophid species, *Benthosema glaciale* and *Notoscopelus elongatus*, showed genetic structuring on finer geographic scales. The observed structure was not consistent with recent suggestions that “resident” populations of myctophids can maintain allopatry despite the mixing of ocean waters. Rather, it indicates that the very rapid speciation characteristic of the Myctophidae is both on-going and detectable using barcodes.

## Introduction

A full assessment of biodiversity in the world’s oceans requires knowledge of the large, complex and under-studied deep-ocean pelagic ecosystems that include a vast array of species and fulfil critical ecological roles [1]. Among the many species, those fishes that spend the daylight hours in the mesopelagic zone, at depths between 200 and 1,000 m, typically undertake diel migrations, feeding in surface waters at night. In contrast, the species of the bathypelagic zone, 1,000 to 4,000 m depth, are mostly non-migratory [2], though some species straddle the boundary between the zones. Many nominal species found in each depth zone are broadly distributed geographically, even circumglobally, while their habitats appear invariant and undivided across vast areas [3]. Yet several orders and families of teleosts found at these depths are highly speciose, with the Myctophidae alone including about 250 recognized species globally [4]. The taxonomy of the deep pelagic fishes remains immature, however, with revisions and new species being published frequently (e.g. [5–14]), while recent research using DNA-based approaches has discovered additional biodiversity in the form of morphologically conserved cryptic species complexes (e.g. [3,4,15,16]). Further confounding understanding of the biodiversity of these fishes, the high cost of mid-ocean research leads to a paucity of sampling, hence also to a scarcity of specialist taxonomic expertise. The limited energy supply to deep pelagic ecosystems [1] results in many species having weak bodies that are easily damaged during capture, often with the loss of diagnostic structures. Meanwhile, some taxa display a variety of other complicating issues, such as paedomorphosis [17] or obscure sexual dimorphism [18]. Morphological identifications of many species can thus be problematic even for specialists, let alone the non-specialists tasked with that responsibility at sea during routine surveys.

First proposed by Hebert and colleagues [19], DNA barcoding generally uses a standard region of the mitochondrial cytochrome c oxidase subunit I (COI) gene as an identifiable characteristic of each species. Barcoding is particularly useful in areas of taxonomy, ecology and biodiversity assessment [3,20,21], by helping in the identification of unknown or uncertain biological material through the comparison of a DNA sequence to a reference set of sequences of known origin. Of particular note here, by focusing the attention of many separate studies on the same gene and so allowing comparisons of sequences from multiple nominal species of fish in different regions, barcoding is proving valuable in highlighting cases of potential cryptic species, for subsequent detailed study (e.g. [3,15,16,22–32]). Evaluation of intra- and inter-species genetic divergence may additionally provide a universal value, the “barcode gap”, a necessary attribute for distinguishing species from higher taxa with such data.

Global biodiversity assessments, including those of deep-pelagic fishes, are assisted by the Barcode of Life Database (BOLD: www.boldsystems.org), GenBank (www.ncbi.nlm.nih.gov/genbank/), FishTrace (https://fishtrace.jrc.ec.europa.eu/) and other international initiatives that compile and catalogue baseline inventories of genetic data, allowing new sequences to be compared through alignment against validated information. Quality-assurance criteria for inclusion of reference sequences in these databases include standards defining “barcode compliance” set out by the Consortium for DNA Barcoding. Those include provision of the name of the species from which the sequence was derived, usually based on morphological identification, a minimum sequence length of 75% of the base pairs (bp) in the barcode region and the provision of two trace files. The location and date of collection of the specimen are recommended additions, which also include specifications for the quality of the sequences. The database validation process involves linking morphological and genetic identifications, measuring identification accuracy and sequence variation within and between species, as well as an evaluation of the utility of DNA barcoding for different taxa.

We here provide two reference data sets of fully compliant DNA barcodes of mesopelagic and upper bathypelagic fishes collected from off the Atlantic coast of Canada. The first set is based on specimens taken in The Gully, a submarine canyon incised into the Scotian Shelf, most of which falls within a Marine Protected Area (MPA), while the second set is based on specimens caught over the continental shelf and slope, from Davis Strait to Georges Bank. Specimens from The Gully were collected by a targeted mesopelagic and bathypelagic survey program, while those from the broader geographic area were collected during routine surveys for commercial fishery resources. Those reference data sets were used to evaluate the presence of the barcode gap and, in combination with publicly-available data from the BOLD database, were reviewed for evidence of potential cryptic species complexes. Specimens from either collection program that could not be fully identified morphologically and/or were missing information required for barcode compliance were compiled into a query data set and identified, based on consensus between multiple genetic approaches: distance- and character-based tree methods, nonlinear-projection algorithms (Multi-Dimensional Scaling: MDS) and non-tree character diagnostics. We show that DNA barcoding is generally successful in discriminating and identifying the species in our data sets. However, we identify multiple cases of potential cryptic species and some examples of other deficiencies in current taxonomy.

## Materials and methods

A diagram illustrating the complex workflow of this study is available as S1 Fig.

### Ethics statement

Approvals to sample fish from the Gully Marine Protected Area were obtained from Fisheries and Oceans Canada’s Maritimes Region Oceans and Coastal Management Division who reviewed the operating procedures and locations prior to undertaking the research. All specimens were taken by government research ships operated by the fisheries agencies of their respective flag states, under licences or permits issued by those agencies and, for specimens taken outside the waters under the jurisdiction of the flag state, under standing bilateral or multilateral arrangements for scientific surveys.

### Sample collection

Mesopelagic and upper bathypelagic teleost specimens (*N* = 557) were collected during 2006–2009 from Atlantic Canadian continental shelves and slopes. The majority of our specimens were collected from The Gully submarine canyon (44°N 59°W) during a four-year study of its pelagic fauna. Surveys of the micronekton and nekton of the MPA were carried out by Canada’s Department of Fisheries and Oceans (DFO), using an International Young Gadoid Pelagic Trawl (IYGPT) and following a depth-stratified, fixed-station design, with the three principal stations aligned along the canyon thalweg, and with replicate sampling in each of daylight and night. Routine trawling extended to 1,750 m depth, though some additional sampling at greater depth (to a maximum of 2,300 m) was undertaken [33,34]. Specimens for DNA barcoding were collected on the surveys conducted during August/September of 2007 and 2008. (All were dead or dying when the nets were hauled aboard.) Following identification to the lowest possible taxon (by an ichthyologist on board), counting, weighing, and measuring, the fish specimens were processed for DNA barcoding. The aim was to select the first one to five specimens of each fish species caught. Selected specimens were given an at-sea identification number, photographed, and then a muscle-tissue sample was extracted and preserved in 95% ethanol. The remainder of each specimen was fixed in formalin before transfer to 95% ethanol, whereupon all were deposited at the Atlantic Reference Centre, St. Andrew’s, New Brunswick (ARC) as voucher specimens. There, they were assigned a number in that collection’s catalogue. Those with uncertain at-sea identifications were re-examined at the ARC by an ichthyologist.

**Fig 1:**
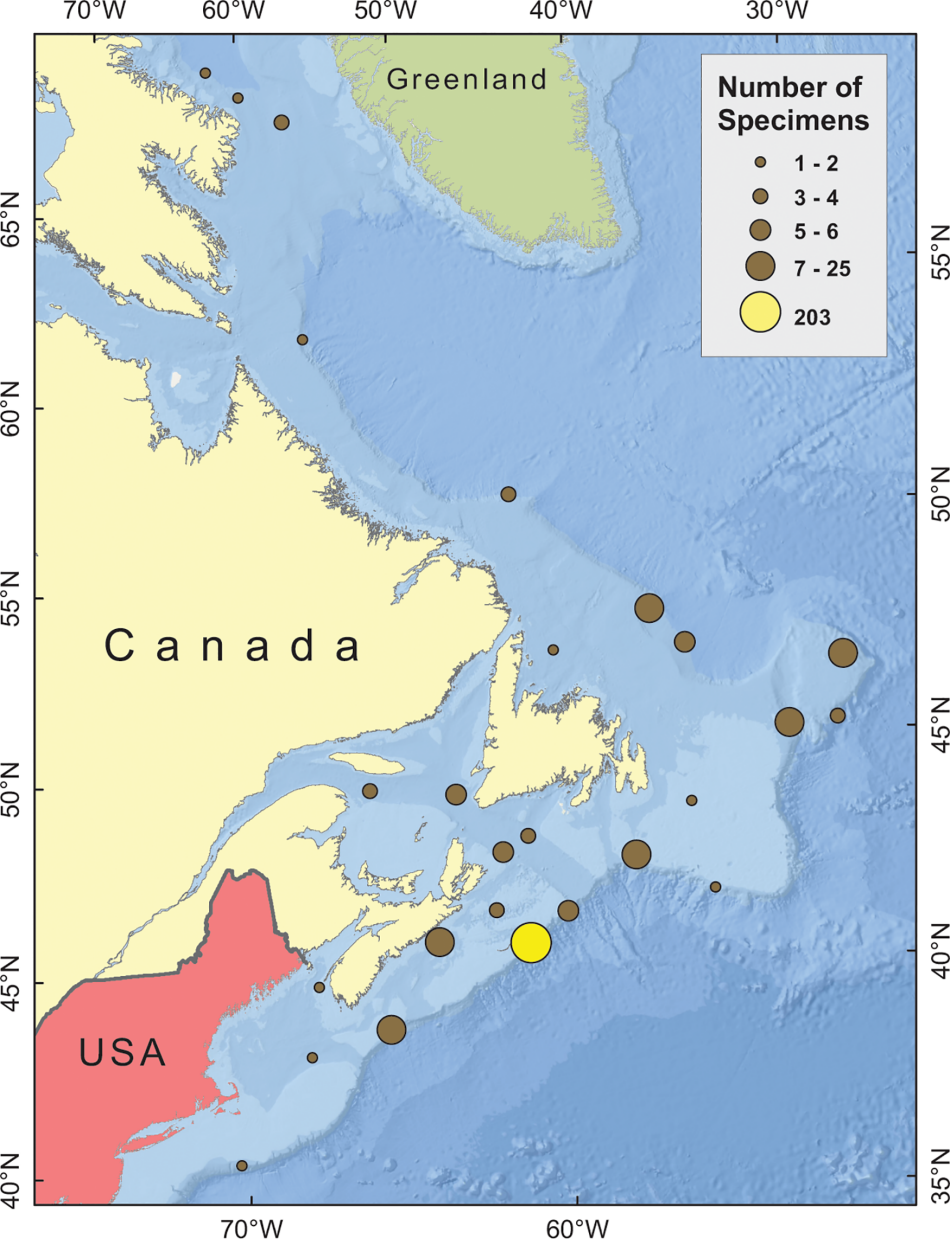
Map showing capture locations of specimens in the reference data set. The yellow dot represents the specimens in the ACMB BOLD project and marks the location of The Gully. The other dots represent the specimens in the ACMF project, ones caught in close proximity being grouped together for clarity. (Projection: Lambert Conformal Conic).

The second collection of specimens was gathered opportunistically between 2006 and 2009 aboard Canadian, European Union and United States survey vessels, using various sampling gears, during a study of the barcodes of demersal fishes [35]. Most were collected between the northern Grand Banks and the Scotian Shelf (northwest Atlantic) though some specimens were taken in Davis Strait and others on Georges Bank (Fig 1). Many of those specimens were in very poor condition, and all were dead, by the time that they were first handled. They were typically preserved frozen, at -5°C, while at sea, with non-specialist identifications attached to each specimen. Most were photographed prior to tissue sampling. Many voucher specimens were deposited at the ARC, after thawing, fixation and preservation in ethanol, but that was not done consistently. Those sampling deficiencies notwithstanding, this second collection was considered important in expanding the spatial coverage of our data and testing the efficacy of our reference data set.

### DNA preparation and sequencing

In the lab, genomic DNA was extracted with a glassmilk protocol [36]. The CO1 region (650 bp) was amplified using primer cocktails and thermocycling protocols designed for barcoding fishes [37]. All specimens were amplified with one or another of the primer combinations from Ivanova and colleagues [37], the exact primers for each specimen being recorded in the BOLD databases for this project. We ran 12.5 μL volume PCRs containing 20 mM Tris-HCl, 10 mM (NH_4_)_2_SO_4_, 10 mM KCl, 2 mM MgSO_4_, 0.1% Triton X-100, 50 μM each dNTP, 0.5 U *Taq* DNA polymerase (New England Biolabs), 0.1 μM primer cocktail and 20–100 ng genomic DNA. Thermal cycling conditions were 94°C for 2 min, 35 cycles of 94°C for 30 s, 52°C for 40 s and 72°C for 1 min, with a final extension at 72°C for 10 min. Polymerase chain reactions (PCRs) were imaged on 1.0% agarose stained with gel-green (Biotium, Hayward, CA, USA), and successful amplifications were sent for sequencing in both directions to either the Canadian Centre for DNA Barcoding (University of Guelph, Ontario, Canada) or Macrogen Inc. (Seoul, Republic of Korea).

Sequence data were submitted to BOLD and GenBank. The sequence for each specimen was then assigned BOLD Process Identification and GenBank reference numbers, in addition to its ARC and at-sea sample numbers.

### Preparation of reference data set

Mitochondrial DNA sequences from specimens that were morphologically identified by a specialist ichthyologist, that had complete associated data (e.g., image, location) and that had high quality genetic data, were aligned using the CLUSTALW ALIGNER in MEGA 6 [38,39] and manually trimmed to 503 bp, except for 9 that were shorter, as the missing data ratios per site were high at the beginning and the end of sequences. FABOX 1.41 [40] was used to collapse sequences into unique haplotypes. Next, the Kimura 2-Parameter (K2P) model of sequence evolution was used to estimate the phylogeny of haplotypes using 1,000 bootstrap replicates of the neighbour joining (NJ) and maximum likelihood (ML) estimations in MEGA. NJ trees were created for each taxonomic order to facilitate inspection for potential errors and inconsistencies between morphological identifications and phylogenetic branch placement. Placement of specimens into orders followed the BOLD taxonomic system. Any specimens that did not fall into expected clades were re-examined for data entry errors by checking the at-sea identification, identification numbers and photographs, with mistakes being corrected before analysis proceeded.

Those resulting sequences which were confirmed to be fully barcode compliant, with voucher specimens deposited at the ARC, were placed in the BOLD “Atlantic Canada Mesopelagic and Upper Bathypelagic Fishes of the Gully MPA” (ACMB) project if they were collected in The Gully submarine canyon (*N* = 247) or in the “Atlantic Canada Mesopelagic Fishes” (ACMF) project if they were collected in the broader geographic area (*N* = 119). Together, those comprised our reference data set (Fig 1, S1 Table).

### Preparation of query data set

In addition to those represented in our reference data sets, another 191 specimens or tissue samples were collected that could not be made fully barcode-compliant. Their barcode sequences comprise a query data set, placed in the BOLD project “Atlantic Canada Mesopelagic Fish Miscellaneous” (ACMM). Of those, 117 specimens were examined by a specialist and deposited at the ARC, but their sequences could not be included in the reference data sets because they did not meet the barcode compliancy standards for one or more reasons (e.g., 41 were too damaged for specific identification). A further 74 tissue samples were collected at sea from specimens that were not retained and hence not deposited as vouchers for verification and future reference. Only at-sea identifications by non-specialists are available for those samples, 24 of which were not identified to species.

### Publicly available BOLD data set

For some analyses, we combined that reference data set with sequences, publicly available in BOLD during October 2016, from all individuals within the taxonomic orders represented in either or both of the reference and query data set. Only the Notacanthiformes, represented by *Aldrovandia affinis*, was present in the query data set but not the reference data set.,. Those sequences were trimmed to 503 bp as described above and compiled as a “publicly available BOLD” (PAB) data set. Addition of the PAB data set provided a global context for analysis of specimen placement, allowing identification of potential cryptic species complexes. However, many of the sequences included in the PAB data set had been mined from GenBank and are not fully barcode compliant. Some lack collection details, while others may introduce error through mistaken morphological identifications and/or laboratory errors that were not detected at the time of submission. Any specimens that did not fall into expected clades were re-examined for the above errors by checking all supporting information, with mistakes being corrected before analysis proceeded.

### Barcode summary statistics and tests of barcode efficiency

Assessment of the barcode gap and calculations of K2P distances within species, genera and families were conducted using the BOLD online sequence analysis tools and the sequences in the merged ACMB and ACMF reference data set. Sequences were aligned using a Hidden Markov Model (HMM) profile of the COI proteins with BOLD Aligner. The within-species distribution was normalized using the BOLD tool to reduce bias in sampling at the species level. Following BOLD convention, sequences with < 2% maximum sequence intra-specific divergence were considered putative species. As a test of barcode efficiency, species monophyly in NJ trees of the ACMB, ACMF and PAB data sets, generated in MEGA, was assessed.

### Tests for cryptic species complexes

NJ and ML trees, created in MEGA using the ACMB, ACMF and PAB data sets, were searched for potential cryptic species complexes. Sister clades within nominal species were considered genetically independent if they met a criterion of reciprocal monophyly with > 70% bootstrap support and contained more than one specimen in each clade. The level of bootstrap support used in this selection was arbitrary.

### Molecular identification of specimens in the query data set

DNA-based specific identifications of specimens that are not identifiable morphologically can only be made if their species are represented in available databases. We employed four approaches to genetically identify specimens in the ACMM data set: (1) K2P nearest neighbour distances in NJ trees, (2) sequence similarity in BOLD, (3) sequence similarity in GenBank, based on BLAST searches performed individually against that database, and (4) characteristic attribute diagnosis using the Character Attribute Organization System (CAOS) [41–43]. The NJ trees were generated in MEGA using the combined reference, PAB and query data sets, ExcaliBAR software was used to calculate nearest neighbour K2P distances [44]. In some instances, NJ tree placement of unnamed or uncertain specimens was cross-checked with that in ML trees using 1,000 bootstrap iterations, MDS plots [45] and the outputs of CAOS analyses [46].

In preparation for application of the CAOS software, we created ML trees in MEGA using the ACMB, ACMF, ACMM and PAB data sets. FASTA files were made of the data for each taxonomic order present in the combined data. Any modification of FASTA file headers was performed in FABOX. The corresponding ML tree and FASTA file were then joined in MESQUITE [46] and exported as a nexus file for use in CAOS.

A DNA-based assignment to species was made when there was agreement among any three of the four identification methods. Where the methods suggested different species of the same genus, only a generic identification was assigned to the specimen.

## Results

### Reference data set

The reference data set generated by this study comprised 366 sequences, which included 328 unique haplotypes and representatives of 118 species, 93 genera, 42 families, and 16 orders (Table 1, S1 Table). Most of those, 247 sequences and representatives of 106 species, were from The Gully (ACMB project). Data from the broader Canadian Atlantic region (ACMF project) comprised 119 sequences, including 12 species, seven genera and two families not found in ACMB (S1 Table). Sample sizes averaged 3.1 individuals per species. However, 43 of the 118 species were represented by only a single specimen, while higher taxa also had low sample sizes. Thus, barcoding effectiveness could not be determined through examination of monophyly. Nevertheless, sequence quality was high throughout. Complete metadata for each sequence were submitted to both BOLD and GenBank. Five species were novel to BOLD: *Bathophilus vaillanti* (Stomiiformes; *N* = 1), *Borostomias mononema* (Stomiiformes; *N* = 1), *Howella sherborni* (Perciformes; *N* = 4), *Laemonema barbatulum* (Gadiformes; *N* = 3), *Paraliparis calidus* (Scorpaeniformes; *N* = 1) and *Oneirodes bradburyae* (Lophiiformes; *N* = 1).

**Table 1:** Summary of reference data set, by taxonomic order.

Order	Specimens	Families	Genera	Species
Anguilliformes	30	6	9	10
Argentiniformes	16	1	3	3
Alepocephaliformes	17	2	6	7
Stomiiformes	75	4	21	26
Aulopiformes	47	6	8	9
Myctophiformes	81	1	15	25
Gadiformes	16	3	4	5
Ophidiiformes	1	1	1	1
Lophiiformes	16	4	7	7
Beryciformes	25	3	6	9
Zeiformes	1	1	1	1
Trachichthyiformes	5	1	1	1
Scorpaeniformes	13	2	2	4
Perciformes	7	4	4	5
Scombriformes	6	2	3	3
Trachiniformes	10	1	2	2
**Total**	366	42	93	118

K2P distance increased with taxonomic level as expected, from a mean (normalized) intra-specific variation of 0.5%, to a mean within-genus inter-specific distance of 13.1%, and mean within-family inter-generic distance of 20.7% (Table 2). A barcode gap was observed for all but one pair of species, *Alepisaurus ferox* and *A*. *brevirostris* (Aulopiformes), amongst the 118, meaning that the maximum intra-specific distance did not exceed the minimum inter-specific distance in any other case (S2 Table). The analysis flagged one other case of low within-genus, inter-specific distance, with only 0.8% divergence between *Scopelogadus beanii* and *S*. *mizolepis* (Beryciformes: S2 and S3 Tables), while four species, including *Alepisaurus ferox*, showed a maximum intra-specific divergence greater than the expected 2% (S2 Table).

**Table 2:** K2P distances within species, genera and families for the 75 species represented by two or more sequences in the reference data set.

	N	Taxa	Number of Comparisons	Minimum Distance (%)	Mean Distance (%)	Maximum Distance (%)	Standard Error of Maximum Distance
Within-Species (W-S)	323	75	791	0.0	0.5	13.1	0.00
Normalized W-S	317	75	-	0.5	0.5	-	0.01
Within-Genus	122	19	237	0.5	13.1	23.9	0.02
Within-Family	297	19	4203	5.8	20.7	34.9	0.00

The lowest within-family inter-generic distance was found in the Platytroctidae (Alepocephaliformes) between *Normichthys operosus* and *Maulisia microlepis*, the sole members of their respective genera represented in the reference data set (mean distance 5.8%: S2 Table). Amongst the Myctophidae (Myctophiformes), the mean distance between *Lampanyctus macdonaldi* and *Nannobrachium atrum* was only 9.1%, which was less than the distances between either of those and their nearest neighbours within their own nominal genera (S2 and S3 Tables).

### Reference and PAB data sets

**Fig 2:**
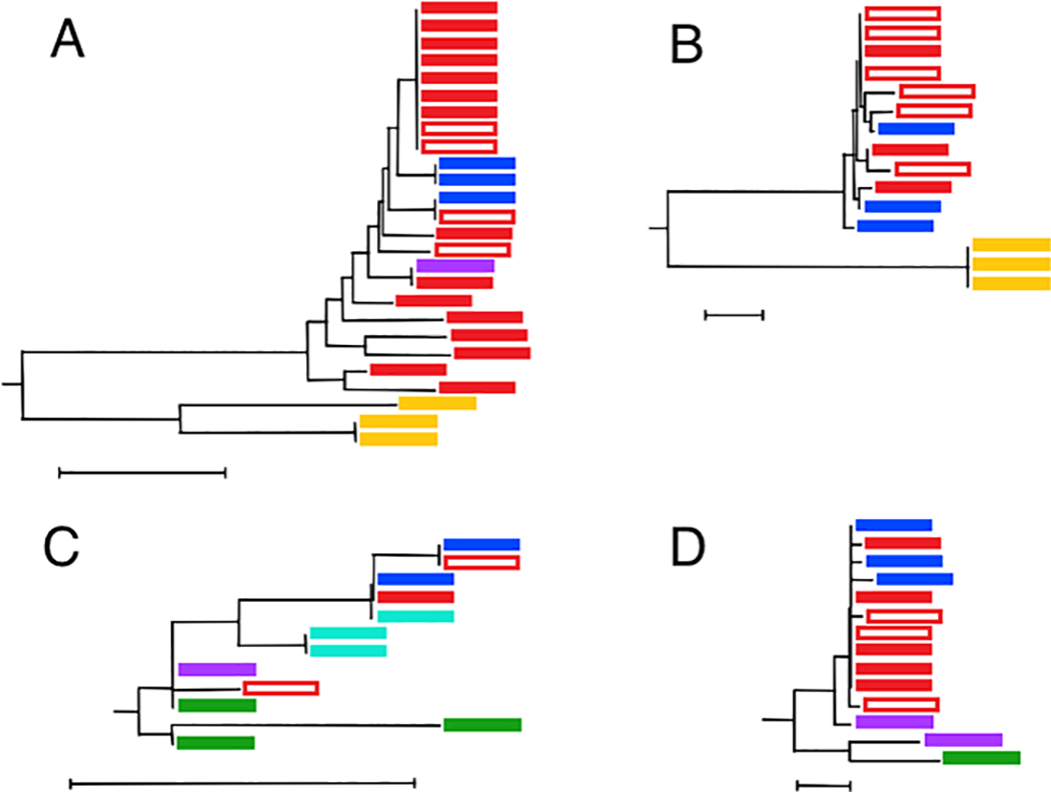
Neighbour-Joining (NJ) trees of sequences in the reference and PAB data sets for selected nominal species, based on Kimura 2-Parameter (K2P) genetic distances. A) *Arctozenus risso*; B) *Chiasmodon niger*; C) *Benthosema glaciale*; D) *Notoscopelus elongatus*. Sequences are colour coded by origin of specimen (red: northwest Atlantic, open rectangle: The Gully; dark blue: Mid-Atlantic Ridge; purple: Greenland; light blue: Jan Mayen; green: Balearic Islands; yellow: northeast Pacific). Duplicate PAB sequences were deleted prior to analysis. Each tree is drawn to scale, with branch lengths in K2P genetic distances. The scales differ among trees but all scale bars represent distances of 0.01. (See S2 Fig for identification of each sequence).

Most of the sequences in the reference data set were placed where expected in both the NJ and ML trees prepared from the combination of that set and the PAB data set. Application of our criteria for identification of potential cryptic species, found 24 examples (Table 3, Fig 2, S2 Fig). One, *Alepisaurus* spp., resulted from a deficiency in standard identification keys, leading to some sequences being assigned to the wrong species. Most of the others suggest possible specific distinctions of populations on ocean-basin scales (e.g. Fig 2A and 2B), though two of the Myctophids (*Benthosema glaciale* and *Notoscopelus elongatus*) appear more finely subdivided (Fig 2C and 2D). Each is discussed below.

**Table 3:** Nominal species containing sister clades with reciprocal monophyly and two or more specimens in each clade in the NJ and ML trees of the reference and pab data sets.

Order	Species	Order	Species
Anguilliformes	*Avocettina infans*	Myctophiformes	*Ceratoscopelus warmingii*
Anguilliformes	*Eurypharynx pelecanoides*	Myctophiformes	*Hygophum hygomii*
Anguilliformes	*Synaphobranchus kaupii*	Myctophiformes	*Lampanyctus photonotus*
Argentiniformes	*Bathylagus euryops*	Myctophiformes	*Lepidophanes guentheri*
Stomiiformes	*Chauliodus sloani*	Myctophiformes	*Nannobrachium atrum*
Stomiiformes	*Polymetme corythaeola*	Myctophiformes	*Notoscopelus elongatus*
Stomiiformes	*Sigmops bathyphilus*	Lophiiformes	*Chaenophryne longiceps*
Aulopiformes	*Alepisaurus ferox*	Lophiiformes	*Cryptopsaras couesii*
Aulopiformes	*Arctozenus risso*	Beryciformes	*Cetostoma regani*
Aulopiformes	*Magnisudis atlantica*	Beryciformes	*Poromitra crassiceps*
Aulopiformes	*Scopelosaurus lepidus*	Perciformes	*Priacanthus arenatus*
Myctophiformes	*Benthosema glaciale*	Trachiniformes	*Chiasmodon niger*

### Species identification through molecular assignment

Our identification approach allowed us to confidently assign genetic-based species names to 156 of the 191 specimens (82%) in the ACMM data set, including 56 that had not been identified to species morphologically. Of the 100 specimens identified to species both morphologically and genetically, the specific identifications agreed in 66 cases. A further 11 specimens were identified to genus. Most of the other 24 specimens were of species not represented in the reference or PAB data sets, while in some cases sequences in the PAB data set appeared to have been derived from misidentified specimens.

## Discussion

The need for strict quality control when assembling reference barcodes for fish is clear [47], especially when the subjects are mesopelagic and bathypelagic species, with the complications of immature taxonomy, scarce specialist expertise and frequent damage to weak specimens. We therefore excluded from our reference data set all sequences that were not fully barcode-compliant and those which lacked a well-found morphological identification. Thus constrained, only a limited number of our reference sequences proved to be discordant and much of that discordance can be explained.

### Deficiencies in identification keys

No barcode gap was evident between the sequences for *Alepisaurus ferox* and *A*. *brevirostris* in our reference data set, while nominal *A*. *ferox* sequences fell within two sister clades when the PAB data set was added (S2 Fig). The standard keys for the genus emphasize the relatively longer snout and head of *A*. *ferox*, compared to those of *A*. *brevirostris*. However, a rarely noted study [48] found those characters unreliable in individuals shorter than about 500 mm standard length. Only one of the five *Alepisaurus* specimens represented in our reference data set (SCAFB1030) was large, at 1050 mm, the others all being shorter than 300 mm. The large individual was identified as *A*. *ferox* and its sequence clustered with others from that species in the PAB data set. In contrast, all of the sequences for the small specimens in the reference data set, including ones identified to each of the species, fell into a second clade, where they were joined by a sequence from a 547 mm specimen of *A*. *brevirostris* taken near Georges Bank. Thus, the observed discordance appears to have arisen from defective keys.

While our identifications were not affected, the supposed diagnostic characters used to distinguish some species within *Synaphobranchus* (Anguilliformes) have also proven deficient [49]. The sequences in our reference and PAB data sets for members of that genus showed four clades (S2 Fig), including a major one composed of specimens from Canadian waters and the Mid-Atlantic Ridge, plus one from off Portugal, all of them identified as *S*. *kaupii*. A second major clade consisted of specimens taken off South Africa which have recently been re-identified as *S*. *affinis*, while there was a single example of *S*. *brevidorsalis* from off British Columbia. The final clade comprised three sequences, which may have all come from one specimen identified as *S*. *kaupii* that was taken off Japan. Whether that represents a cryptic species or a further example of identification deficiency remains unclear.

### Deficiencies in morphologically based taxonomy

Within the reference data set, the myctophids *Lampanyctus macdonaldi* and *Nannobrachium atrum* proved to be more similar than either was to its nearest neighbour within its own nominal genus. Their tribe, the Lampanyctini, has long posed challenges for taxonomists [50]. Zahuranec grouped 17 species into *Nannobrachium* on the basis of their shared possession of six morphological characteristics, in the process moving *N*. *atrum* and *N*. *lineatum* (the species of *Nannobrachium* in the reference data set) out of *Lampanyctus* [51]. However, a recent molecular phylogenetic study, using a combination of nuclear and mitochondrial markers, found the two genera intermingled. *L*. *macdonaldi* and *N*. *atrum* clustered together, while *N*. *lineatum* appeared as the sister taxon to a clade containing most other members of both genera. *Nannobrachium* will presumably have to be merged back into *Lampanyctus* [52].

Examination of our reference data set found the distance between *Scopelogadus beanii* and *S*. *mizolepis* (Beryciformes) to be less than that of the expected barcode gap. An earlier analysis of data extracted from BOLD (which did not include our ACMB and ACMF projects) likewise found those two species to be “barcode-indistinguishable” [52]. The two forms are morphologically closely similar and have overlapping ranges: in the western Atlantic, *S*. *beanii* has been reported from the Gulf of Mexico to Greenland, whereas *S*. *mizolepis* is known from Brazil to the Grand Banks [53]. It is possible that the nominal species are not biologically distinct or alternatively that they separated through a recent speciation event, not yet fully reflected in their barcode sequences.

Our data also indicated that the platytroctids *Normichthys operosus* and *Maulisia microlepis* (Alepocephaliformes) are so similar as to suggest a congeneric relationship. That was additional evidence of the known weakness of the existing taxonomy of the Platytroctidae. Recent whole-mitogenome studies have begun to resolve the issues but have not yet addressed fine distinctions between genera [54,55].

### Potential basin-scale cryptic species

The mesopelagic and bathypelagic zones of the world ocean appear to offer vast horizontal extents of relatively invariant habitats, with few evident physical barriers to gene flow. Many of the fishes living in those zones have been assigned, on morphological grounds, to nominal species that are broadly distributed, even circumglobal [3]. Yet, some early studies suggested fine, intra-specific morphological distinctions among populations in different ocean basins (e.g. [56]). The advent of molecular-phylogenetic techniques demonstrated that some of the nominal species are actually cryptic complexes, including ones with multiple species in the same ocean (e.g. [57]). The extent of such complexity has been illustrated by a recent study of nominal fish species with circumglobal distributions in tropical and warm-temperate latitudes. That found 284 examples, of which 200 are pelagics living below 200 m depth. Only 52 of the latter (10 of them represented in our reference data set) had sufficient barcodes in BOLD for analysis, at the time that the data were extracted (2014–15), but 20 of those 52 appeared to be species complexes [3]. Similarly, inspection of the trees built from our combined reference and PAB data sets found 22 cases of potential cryptic species for which closer taxonomic study may prove useful (the 24 listed in Table 3, less those which met our criteria through identification error: *Alepisaurus ferox* and *Synaphobranchus kaupii*), a list only partially congruent with the earlier work [3].

Among the 22, 13 suggest differentiation between members of the same nominal species in different ocean basins. Those with possible distinction between fish in the Atlantic and Pacific include: *Avocettina infans* (Anguilliformes: S2 Fig; distinction previously noted by [3]), *Arctozenus risso* (Fig 2A, S2 Fig), the Atlantic clade of which was internally variable, *Magnisudis atlantica* (Aulopiformes: S2 Fig) and probably *Cryptopsaras couesii* (Lophiiformes: S2 Fig).

The available sequences for *Ceratoscopelus warmingii* (Myctophiformes) form three clades, one containing specimens from the western North Atlantic, one with fish from French Polynesia and the third containing a specimen from the coast of South Africa and two others from American Samoa (S2 Fig). The genus has long posed challenges for taxonomists. Besides *C*. *maderensis* in the North Atlantic, which is a clearly distinct species, the genus comprises a *townsendi*–*warmingii* complex. That is conventionally recognized as containing a cosmopolitan species, *C*. *warmingii*, and a northeast Pacific congener, *C*. *townsendi*, but some authors have suggested multiple, partially isolated units within a single species [56]. Recent molecular phylogenetic studies have found four major clades within the genus [50], though finer-scale genetic structure has been detected in the South Atlantic [58].

*Hygophum hygomii* (Myctophiformes) shows one clade from the North Atlantic and another from the western Mediterranean (S2 Fig). Distinctions between individuals in the North Atlantic and their nominal conspecifics either off South Africa, in the South Atlantic or in the equatorial Atlantic are suggested for *Polymetme corythaeola* (Stomiiformes: S2 Fig), the myctophids *Lepidophanes guentheri* (which shows two clades in the tropical Atlantic, plus one in the north, besides two sequences from the Caribbean) and *Nannobrachium atrum* (S2 Fig), plus *Priacanthus arenatus* (Perciformes: S2 Fig). The latter is a species of Atlantic warm-water reefs. An expatriate juvenile specimen taken in The Gully clustered very closely with several specimens from the coast of Brazil and one caught off Alabama, USA, while three sequences from off the Indian Ocean coast of South Africa formed a second clade. *Lampanyctus photonotus* (Myctophiformes: S2 Fig) may be another example of basin-scale differentiation, in that one of its clades comprises specimens from the Mid-Atlantic Ridge in the equatorial zone and the South Atlantic, plus one individual from the Sargasso Sea, whereas the other clade contains one specimen from The Gully and two from the Mid-Atlantic Ridge near 42° 45' N.

Two related examples are *Chiasmodon niger* (Trachiniformes) and *Poromitra crassiceps* (Beryciformes), though in their cases specific distinctions have already been suggested, albeit not yet fully implemented in databases and field sampling programs. *Chiasmodon* has recently been the subject of two contrasting reviews [11,14]. Both agreed that most of the individuals of the genus in and under the sub-tropical gyre of the North Atlantic are properly named *C*. *niger*. However, one placed all *Chiasmodon* outside the western Pacific into that one species [14], whereas the other saw seven species in the genus, including distinguishing those in the easternmost Pacific as *C*. *subniger* and erecting a new species, *C*. *harteli*, for a form primarily found in the North Atlantic sub-polar gyre [11]–conclusions that have been disputed [12,13]. The distinction seen in the barcode sequences was between three specimens taken off California, USA and 12 caught in the North Atlantic (Fig 2B, S2 Fig). Thus, it is fully in accord with one of the conclusions of M.R.S. Melo [11], if the Pacific specimens were members of his *C*. *subniger*. The available barcode sequences from the North Atlantic fall into a single clade. It remains unclear whether that indicates the lack of a specific distinction between *C*. *niger* and *C*. *harteli* or, alternatively, that only members of a distinct *C*. *harteli* have yet contributed barcode sequences. Of the 12 available, six were from specimens taken in The Gully and one from the continental slope 240 km further west. While the waters in the canyon are primarily sub-polar (surface waters derived from the Gulf of St. Lawrence and the Labrador Current, with Labrador Sea Water at depth [59]), the margin of the sub-tropical gyre at the surface was only ≈10 km from the southernmost sampling station during the 2007 survey, when much of the deep water in the canyon appears to have been derived from sub-tropical origins, having passed under the Gulf Stream [34, 59]. Thus, either or both forms of *Chiasmodon* might be present. There was one other sequence from Flemish Cap (easternmost of the Grand Banks), one from northeast of Newfoundland and three from the Mid-Atlantic Ridge between 52° 45' and 53° 11' N. The North Atlantic Current, forming the shared limb of the two gyres, is topographically constrained to cross the Ridge *via* the Charlie Gibbs and Faraday fracture zones [60,61], and hence specimens taken north of 52° N were likely from the waters of the sub-polar gyre. In all those areas, *C*. *harteli* would be the expected species, if it is indeed distinct.

*Poromitra* has recently been substantially revised, with descriptions of multiple new species and reassignment of names. The genus is now suggested to comprise 23 species globally [5–10]. The available barcode sequences mostly form separate North Atlantic and northeast Pacific clusters (S2 Fig), corresponding respectively to the ranges now suggested for *P*. *nigriceps*, plus perhaps *P*. *megalops*, and *P*. *cristiceps*, plus *P*. *rugosa* [5,7,10]. However, both clades are internally variable, the Atlantic one including an aberrant sequence from The Gully and a sequence from a Pacific specimen. Clearly, more study is needed to reconcile the barcode results with the new morphologically based taxonomy.

The pattern of one species living in the sub-tropical gyre of the North Atlantic and a congener in the sub-polar gyre, suggested for *Chiasmodon niger* and *C*. *harteli*, is perhaps implied by three further examples of potential cryptic species, though each shows additional complications. The available sequences for *Scopelosaurus lepidus* (Aulopiformes) show two clades in the North Atlantic (S2 Fig), one containing two sequences from The Gully and two from the Mid-Atlantic Ridge south of 43° 10' N–so far south as to be in a distinct Azorean faunal zone for mesopelagic fishes [62,63]. The other clade contains one specimen from each of Bear Seamount (south of Georges Bank and within the sub-tropical gyre), Flemish Cap, the Mid-Atlantic Ridge at 52° 45' N and Greenland. Those two clades are not sisters. The former clusters with the North Pacific species *S*. *harryi* and the latter with a single sequence from *S*. *hamiltoni*, a species of the Southern Ocean. The Flemish Cap sequence (our SCAFB1003-07) differed sufficiently from those obtained from the Gully specimens that the mean and maximum intra-specific distances in the reference data set were 6.2% and 9.3% respectively.

The sequences for *Eurypharynx pelecanoides* available in BOLD show two clades, each containing a member from the Mid-Atlantic Ridge, plus a lone sequence from the eastern North Pacific (S2 Fig). Two specimens from The Gully and one from Greenland cluster with one Mid-Atlantic Ridge specimen that was taken north of 56°N, while the other clade includes two specimens taken on the Ridge south of 51° 30' N (somewhat further north than expected for the sub-tropical gyre), one from Bear Seamount and one from off Japan. Of note, this structure within *E*. *pelecanoides* was not apparent to [3], only emerging with the addition of the Greenland and Gully sequences.

Similarly, *Sigmops bathyphilus* (Stomiiformes) shows two clades, one containing two specimens taken on the Mid-Atlantic Ridge north of 55° 30' N, the other having two taken on the Ridge south of 53° 10' N (S2 Fig). The two specimens from The Gully clustered with the northern clade.

Four final cases might involve cryptic species but ones with less clear-cut geographic relationships than those considered above. The available sequences attributed to *Chauliodus sloani* (Stomiiformes) mostly form a single clade, presumably representing that species, which included four sequences from our reference set (S2 Fig). However, variation within the nominal species has previously been noted [3] and our sequence SCAFB828 was so aberrant that it raised the mean and maximum intra-specific distances for the *C*. *sloani* in the reference data set to 5.4 and 13.1%, respectively, whereas exclusion of that one specimen reduced the maximum to 0.9%. When the reference and PAB data sets were combined, sequence SCAFB828 closely matched a sequence drawn from a specimen collected off Japan. Of the nine recognized species in the genus, only *C*. *sloani* is expected to have a distribution encompassing both the northwest Pacific and the northwest Atlantic, while only *C*. *sloani* and *C*. *macouni* (five sequences from which formed another discrete clade) have known ranges that include the waters off Japan. Thus, the clade containing SCAFB828 suggests an undescribed species with broad distribution.

*Cetostoma regani* (Beryciformes) also shows internal structure, as had previously been noted [18]. The available sequences form two clades with at least one specimen from the North Atlantic in each (S2 Fig). Both sequences from The Gully cluster with one from Bear Seamount and another from the Mid-Atlantic Ridge taken near 42° 50' N. The other clade contains a specimen from the Sargasso Sea, one from the Strait of Florida (identified in BOLD as *Parataeniophorus gulosus*–a name formerly applied to a juvenile form [18]) and another collected off Japan.

The available sequences for *Bathylagus euryops* (Argentiniformes) form two very tight clades, though ones not sufficiently distant from one another as to indicate distinct species (S2 Fig). One clade contained mostly sequences from specimens taken on the Mid-Atlantic Ridge, between 41° 44' and 53° 5' N, though also one each from The Gully, Flemish Cap and off Greenland. The other comprised three sequences from The Gully, three from off Labrador, one from the Davis Strait and two from off Greenland, but lacked representation from the Mid-Atlantic Ridge. Lastly, *Chaenophryne longiceps* (Lophiiformes) may form two or more clades but there are too few known sequences for firm conclusions (S2 Fig). The sole Gully specimen clustered loosely with one from the northeast Pacific, while specimens from Davis Strait and the Mid-Atlantic Ridge near 50° N had near-identical barcode sequences.

The absence of firm physical barriers to range expansion for deep-sea species, especially before the rise of the Panama isthmus cut low-latitude communication between the Atlantic and the eastern Pacific (≈ 9 M years ago at bathypelagic depths but ≈ 4 M years for larvae in surface waters [64]), has allowed many pelagic fishes to become very widely distributed, even circumglobal. The comparatively weak horizontal gradients in environmental gradients have permitted those species to maintain evolutionary fitness in multiple ocean basins without apparent morphological specialization. In consequence, ichthyologists have tended to recognize wide-ranging nominal species–including the 200 supposedly circumglobal pelagics living below 200 m depth [3]. In contrast, the results of the present study, added to other recent work (e.g. [3, 18]), suggest that gene flow has not been maintained in some, perhaps many, such species and that morphological conservatism is concealing speciation of populations in various basins–which has been followed in some cases by re-invasions by members of new clades. How extensive the fragmentation of nominal species of mesopelagic and bathypelagic fishes may be will only become clear as more gene sequences, nuclear as well as mitochondrial, are gathered. Sequences from hundreds of specimens, each accompanied by an expert morphological identification, may be needed before the variations within a single, broadly distributed nominal species could be fully mapped.

### Fine-scale structure in the Myctophidae

The Myctophidae are estimated to have appeared in the Late Cretaceous, some 104 to 73 million years ago. Since then, they have radiated into about 250 nominal extant species, in 33 genera–at about double the speciation rate typical of the Actinopterygii, despite the scarcity of apparent physical barriers to gene flow, or of much horizontal variation in environmental conditions, in their open-ocean habitats [4,65]. Meanwhile, it has long been suspected that bioluminescent fishes use their photophores in species recognition [66], perhaps through the timing of light flashes, in addition to photophore locations, and potentially extending to the sort of complex bioluminescent courtship “dialogues” seen in fireflies [67,68]. While no myctophid mating, nor the associated use of light, has ever been observed, it has recently been argued that the rates of speciation in various groups of myctophids (and of other families in comparison to the Myctophidae) are correlated to the complexity of the patterns of their lateral photophores, suggesting that recognition of those patterns, during mate selection, creates swiftly-evolving behavioural barriers to gene flow [65]. That hypothesis supposes, first, that populations can be effectively isolated from their conspecifics over enough generations that photophore pattern can evolve in allopatry into a distinctive new arrangement and, second, that the Myctophidae have developed a very effective linkage between an individual’s own photophore pattern and the one that it seeks in a mate (though may species are sexually dimorphic in photophore arrangements [65,68]). However, without denying the role of photophores in species recognition, subsequent work has shown that evolutionary change in their locations is primarily a passive consequence of change in body shape, and hence connected to locomotion rather than being driven by potential advantages of genetic isolation [69]–though that cannot negate the possibility of an isolating mechanism based on the timing of flashes, rather than photophore positions (cf. [67]).

Recent molecular phylogenetic studies have found intra-specific genetic structure, expressed over geographic scales much smaller than ocean basins, in some myctophids, including *Ceratoscopelus warmingii*, *Lobianchia dofleini* and *Notoscopelus resplendens* [16,58,70], each of which was represented in our reference data set. None of those three showed comparable structure in the data analyzed here, through *C*. *warmingii* revealed three distinct clades (see above), while *N*. *resplendens* showed a maximum intra-specific distance of 2.3% in the reference data set, indicative of slightly more than expected variation, an anomaly also noted by [3]. Fine-scale structure was, however, evident in *Benthosema glaciale* and perhaps *Notoscopelus elongatus*.

*Benthosema glaciale*, the principal myctophid of the North Atlantic sub-polar gyre and the Norwegian Sea, has abundant populations in Norwegian fjords that are effectively reproductively isolated, both from each other and from the offshore population, by shallow fjord sills and the continental shelf [71,72]. While no genetic data are available, the *B*. *glaciale* in the Mediterranean Sea differ in morphology from those in the northern Atlantic but share some distinctive meristic characteristics with a population on the continental slope off northwest Africa [73], whence individuals could be carried in the Mediterranean Outflow Water. Whether the fjord populations or that in the Mediterranean have developed modified species-recognition signals remains unknown. However, it is possible that fjord-derived (or Mediterranean-derived) lineages could remain partially genetically isolated from strictly oceanic lineages after re-entering the open Atlantic through rare exchange events, at least sufficiently so for multiple mitochondrial lineages to persist.

The available barcode sequences from specimens of *B*. *glaciale* suggest the existence of multiple clades, and hence multiple surviving lineages, but ones too similar to justify specific distinction (Fig 2C, S2 Fig). One Gully specimen clustered with individuals from the Mid-Atlantic Ridge, Davis Strait and Svalbard (plus a specimen of unknown origin), while the second clustered with a specimen from Greenland and two from the western Mediterranean. A third distinct clade contained two specimens from off Jan Mayen Island, plus one perhaps taken off Newfoundland, while a lone individual from the western Mediterranean formed a fourth clade. Some genetic structure within *B*. *glaciale* has been noted previously [15] but only at an ocean-basin scale, the few then-available sequences preventing observation of finer scales.

The standard inventories of marine species recognize two forms within *Notoscopelus*, under the names *elongatus* and *kroyeri*, but disagree as to whether they should be regarded as subspecies or elevated to specific status, BOLD opting for the former. By that nomenclature, *N*. *elongatus elongatus* occurs in the western basin of the Mediterranean Sea, whereas *N*. *elongatus kroyeri* is found across the North Atlantic. The sequences from The Gully, other Canadian waters and the Mid-Atlantic Ridge formed a single, loose clade, presumably representing *N*. *elongatus kroyeri*, and were joined by one sequence from a specimen caught off Greenland (Fig 2D, S2 Fig). A single sequence from the Mediterranean, hence presumably *N*. *elongatus elongatus*, was sufficiently different from the Atlantic clade as to suggest a barcode gap and hence a specific distinction. However, the sister to the Mediterranean example was a sequence from a second specimen taken off Greenland–indicating the presence of a third form, found in the Atlantic but more similar to the Mediterranean species.

Working with sequences from South Atlantic myctophid specimens, N.V. Gordeeva found correlation between the genetic and geographic distances separating the clades within each species that showed fine-scale structure, though the geographic expression of the genetic variation was mosaic-like, rather than clinal. She suggested that the species in question are “residents”, able to maintain their position relative to oceanographic features, thus maintaining genetic isolation [70]. The existence of multiple, closely-related clades within *Benthosema glaciale*, each with broadly overlapping geographic ranges (two of them represented within the confines of The Gully) is inconsistent with that “residency” hypothesis and calls for a different explanation. We suggest that the mechanisms which have led to rapid speciation in the Myctophidae, likely involving intermittent allopatric isolation and mate selection through recognition of photophore patterns [65], are on-going and that they generate multiple sympatric clades best understood as emerging, incipient species.

## Conclusions

The utility of DNA barcoding in identification of unknown specimens, provided that fully barcode-compliant reference sequences of all relevant species are available, has been confirmed as extending to the challenging mesopelagic and bathypelagic fishes. We have demonstrated the need for having robust reference data sets for cataloguing the biodiversity of such fishes–assets that will also enable emerging approaches, such as environmental DNA sampling and gut content analyses [47,74,75], which will be of value to ecosystem based management. While they cannot alone suffice as a foundation for defining species, barcodes have also been shown to be a valuable tool in identifying cases of potential cryptic speciation and other taxonomic complications that merit closer, specialist examination. For the latter purpose to be fully achieved, however, it will not be sufficient to have only a few sequences from each nominal species. Rather, where open-ocean animals are concerned, sequences should be obtained from every ocean basin and every gyre system in which each nominal species occurs, with sufficient individuals represented that the sympatric presence of multiple clades could be detected, if present.

## Supporting information

S1 Fig Flow chart illustrating the workflow of this study. (JPG)

S2 Fig Neighbour-Joining (NJ) trees, based on K2P genetic distances, containing all sequences from the Atlantic Canada Mesopelagic and upper bathypelagic marine fishes reference data set (BOLD Projects ACMB and ACMF) and the publicly available BOLD (PAB) data set for each of the 24 species or genera which showed anomalies in their clades. Branch lengths are scaled to genetic distance. The percentages of bootstrapped replicate trees in which the relevant sequences clustered together are shown above each branch. Notes on the origin of each specimen area annotated. A) *Avocettina infans*; B) *Eurypharynx pelecanoides*; C) *Synaphobranchus kaupii*; D) *Bathylagus euryops*; E) *Chauliodus* spp.; F) *Polymetme corythaeola*; G) *Sigmops bathyphilus*; H) *Alepisaurus* spp.; I) *Arctozenus risso*; J) *Magnisudis atlantica*; K) *Scopelosaurus Lepidus*; L) *Benthosema glaciale*; M) *Ceratoscopelus warmingii*; N) *Hygomphum hygomii*; O) *Lampanyctus photonotus*; P) *Lepidophanes guentheri*; Q) *Nannobrachium atrum*; R) *Notoscopelus elongatus*; S) *Chaenophryne longiceps*; T) *Crypotopsaras couesii*; U) *Cetostoma regain*; V) *Poromitra crassiceps*; W) *Priacanthus arenatus*; X) *Chiasmodon niger*.
(DOCX)

S1 Table List of sequences in the Atlantic Canada mesopelagic and upper bathypelagic marine fishes BOLD projects ACMB and ACMF. (DOCX)

S2 Table Mean and maximum intra-specific K2P genetic distances and nearest neighbour inter-specific distance for each species in the Atlantic Canada mesopelagic and upper bathypelagic marine fishes reference data set (BOLD projects ACMB and ACMF). (DOCX)

S3 Table K2P genetic distances between individuals for each intra-generic, inter-specific pair of sequences in the Atlantic Canada mesopelagic and upper bathypelagic marine fishes reference data set (BOLD projects ACMB and ACMF). (DOCX)
