# Supplementary figures and images for: Barcoding Atlantic Canada’s mesopelagic and upper bathypelagic marine fishes
Source: PLoS One. 2017 Sep 20;12(9):e0185173. doi: 10.1371/journal.pone.0185173 (PMC5607201; doi:10.1371/journal.pone.0185173)

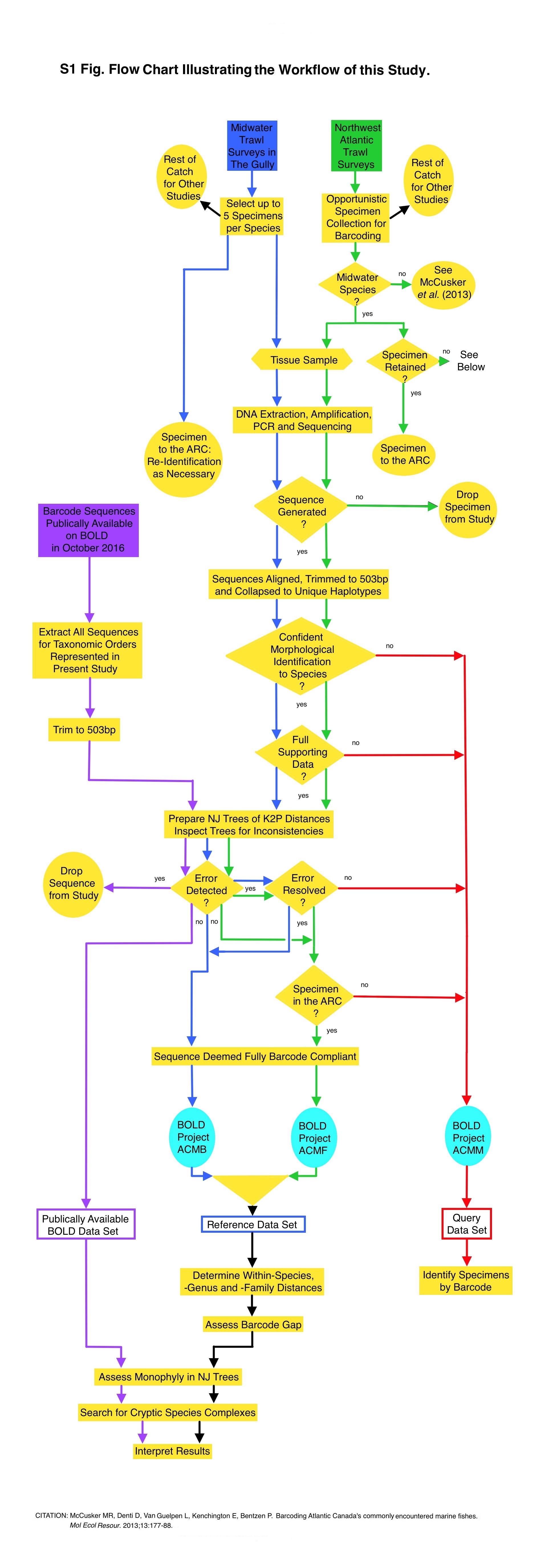

Supplement: S1 Fig — (JPG) [file pone.0185173.s001.jpg]
