# Supplementary material for: Barcoding Atlantic Canada’s mesopelagic and upper bathypelagic marine fishes
Source: PLoS One. 2017 Sep 20;12(9):e0185173. doi: 10.1371/journal.pone.0185173 (PMC5607201; doi:10.1371/journal.pone.0185173)
Supplement: S2 Fig — Branch lengths are scaled to genetic distance. The percentages of bootstrapped replicate trees in which the relevant sequences clustered together are shown above each branch. Notes on the origin of each specimen area annotated. A) Avocettina infans; B) Eurypharynx pelecanoides; C) Synaphobranchus kaupii; D) Bathylagus euryops; E) Chauliodus spp.; F) Polymetme corythaeola; G) Sigmops bathyphilus; H) Alepisaurus spp.; I) Arctozenus risso; J) Magnisudis atlantica; K) Scopelosaurus Lepidus; L) Benthosema glaciale; M) Ceratoscopelus warmingii; N) Hygomphum hygomii; O) Lampanyctus photonotus; P) Lepidophanes guentheri; Q) Nannobrachium atrum; R) Notoscopelus elongatus; S) Chaenophryne longiceps; T) Crypotopsaras couesii; U) Cetostoma regain; V) Poromitra crassiceps; W) Priacanthus arenatus; X) Chiasmodon niger. (DOCX) [file pone.0185173.s002.docx]

**S1 Fig. Neighbour-Joining (NJ) Trees, based on K2P genetic distances, containing all sequences from the Atlantic Canada Mesopelagic and Upper Bathypelagic Marine Fishes Reference Data Set (BOLD Projects ACMB and ACMF) and the Publicly Available BOLD (PAB) Data Set for each of the 24 species or genera which showed anomalies in their clades.** Branch lengths are scaled to genetic distance. The percentages of bootstrapped replicate trees in which the relevant sequences clustered together are shown above each branch. Notes on the origin of each specimen area annotated. A) *Avocettina infans*; B) *Eurypharynx pelecanoides*; C) *Synaphobranchus kaupii*; D) *Bathylagus euryops*; E) *Chauliodus* spp.; F) *Polymetme corythaeola*; G) *Sigmops bathyphilus*; H) *Alepisaurus* spp.; I) *Arctozenus risso*; J) *Magnisudis atlantica*; K) *Scopelosaurus Lepidus*; L) *Benthosema glaciale*; M) *Ceratoscopelus warmingii*; N) *Hygomphum hygomii*; O) *Lampanyctus photonotus*; P) *Lepidophanes guentheri*; Q) *Nannobrachium atrum*; R) *Notoscopelus elongatus*; S) *Chaenophryne longiceps*; T) *Crypotopsaras couesii*; U) *Cetostoma regain*; V) *Poromitra crassiceps*; W) *Priacanthus arenatus*; X) *Chiasmodon niger*.

The species names shown in the trees are those that were attached to the corresponding sequences at the time that the data were downloaded from BOLD. Some appear to have been misidentifications.

These trees include all sequences presented in BOLD when the data were downloaded. Some (identified in the notes) are duplicates. The trees presented in the published Fig 2 have all duplicates eliminated and hence the two trees for *Benthosema glaciale* (Fig 2C and S1L Fig) and those for *Notoscopelus elongatus* (Fig 2C and S1R Fig) differ in detail.

A)

Origins of Sequences:

SCAFB: This study

MAECO: Mid-Atlantic Ridge (BOLD record)

MFC: Off California (BOLD record)

TZFPB: Off British Columbia (BOLD record)

B)

Origins of Specimens:

SCAFB: This study

GLF: Off Greenland (BOLD record)

MAECO: Mid-Atlantic Ridge (BOLD record)

MFC: Off California (BOLD record)

GBGC0016-06: GenBank AB046473:

GBGC1665-06: GenBank NC005299 (derived from AB046473)

Inoue JG, Miya M, Tsukamoto K, Nishida,M. Evolution of the deep-sea gulper eel mitochondrial genomes: large-scale gene rearrangements originated within the eels. Mol Biol Evol. 2003;20: 1917-1924.

That study included five specimens of *E. pelecanoides*, one from Hyuga-Nada, the sea area south of Honshu and east of Kyushu, Japan (about 32°N; voucher specimen: CBM-ZF 10311), and the other four in the equatorial central Pacific (CBM-ZF 10312, 10313, 10316 & 10317). The AB046473 mitogenome sequence was derived from the specimen taken off Shikoku Island, central Japan (Miya, *pers.comm.*).

GBGCA385-10: GenBank FJ918905:

DeVaney SC. Interrelationships of fishes of the order Stomiiformes. PhD Thesis.

The specimen was caught over Bear Seamount (DeVaney *pers.comm*.)

C)

Origins of Specimens:

SCAFB, SCFAC, SCFAD: This study

ABFJ: Off Japan (BOLD record)

DSFSF: Off South Africa (BOLD record)

FCFPS: Off Portugal (BOLD record)

MAECO: Mid-Atlantic Ridge (BOLD record)

TZFPA: Off British Columbia (BOLD record)

As of May 2017, GBGC1681 and GBGC0397 have been deleted from BOLD and their origins are unknown. They may be from the same specimen as ABFJ130.

As of May 2017, all of the DSFSF specimens shown in this tree have been re-identified as *S. affinis*. The TZFPA specimen has been re-identified as *S. brevidorsalis*.

D)

Origins of Specimens:

SCAFB, SCFAC, SCFAD: This study

GLF: Off Greenland (BOLD record)

MAECO: Mid-Atlantic Ridge (BOLD record)

E)


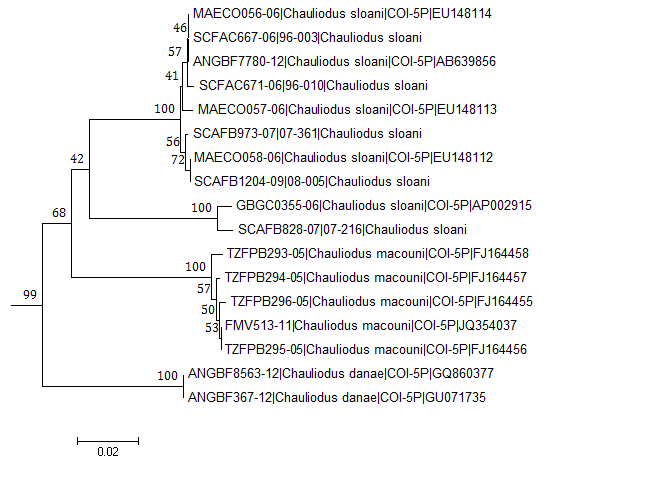


Origins of Specimens:

SCAFB, SCFAC: This study

ANGBF367-12: Sargasso Sea (BOLD record)

FMV: Off Oregon (BOLD record)

MAECO: Mid-Atlantic Ridge (BOLD record)

TZFPB: Off British Columbia (BOLD record)

ANGBF7780-12: GenBank AB639856:

Yanagimoto T, Hoshino K. Database of fishes collected by mid water trawl in the South Indian Sea. [Unpublished and not examined during this study]

ANGBF8563-12: GenBank GQ860377

DeVaney SC. Interrelationships of fishes of the order Stomiiformes. PhD Thesis.

The specimen was probably caught over Bear Seamount (DeVaney *pers.comm*.)

GBGC0355-06: GenBank AP002915:

Miya M, Kawaguchi A, Nishida M. Mitogenomic exploration of higher teleostean phylogenies: a case study for moderate-scale evolutionary genomics with 38 newly determined complete mitochondrial DNA sequences. Mol Biol Evol. 2001;18: 1993-2009.

Specimen taken off Shikoku Island, central Japan (Miya, *pers.comm.*).

BOLD also contains a sequence ANGBF8538-12 (GQ860378) that is identified as *C. sloani*. However, that sequence clustered loosely with one from a specimen identified as *Phosichthys argenteus* and far from other *Chauliodus*.

F)

Origins of Specimens:

SCAFB: This study

DSFSF: Off South Africa (BOLD record)

FCFPI: Off Portugal (BOLD record)

GLF: Off Greenland (BOLD record)

GBGCA357-10: GenBank FJ918933:

DeVaney,S.C. Interrelationships of fishes of the order Stomiiformes. PhD Thesis.

Specimen probably caught over Bear Seamount, where most of DeVaney’s were taken.

G)

Origins of Specimens:

SCAFB: This study

MAECO: Mid-Atlantic Ridge (BOLD record)

H)

Origins of Specimens:

SCAFB: This study

ABFJ: Off Japan (BOLD record)

DSFSF: Off South Africa (BOLD record)

FMV: Off Oregon (BOLD record)

GBGC6637-09: GenBank EU366543:

GBGC6638-09: GenBank EU366542:

Davis MP. Evolutionary relationships of the Aulopiformes (Euteleostei: Cyclosquamata): a molecular and total evidence approach. In: Nelson JS, Schultze H-P, Wilson MVH. (Eds.) ORIGIN AND PHYLOGENETIC INTERRELATIONSHIPS OF TELEOSTS. Verlag Dr. Friedrich Pfeil, Munchen, Germany (in press 2010). [cited source not examined during this study]

GBGCA348-10: GenBank FJ918942:

DeVaney SC. Interrelationships of fishes of the order Stomiiformes. PhD Thesis.

Specimen likely from Bear Seamount, where most of DeVaney’s specimens were taken.

I)

Origins of Specimens:

SCAFB, SCFAC, SCFAD: This study

FMV: Off Oregon (BOLD record)

GLF: Off Greenland (BOLD record)

MAECO: Mid-Atlantic Ridge (BOLD record)

MFC: Off California (BOLD record)

TZFPA: Off British Columbia (BOLD record)

J)

Origins of Specimens:

SCAFB, SCAFC: This study

GLF: Off Greenland (BOLD record)

MAECO: Mid-Atlantic Ridge (BOLD record)

MFC: Off California (BOLD record)

GBGC5908-08: GenBank EU520654:

Gruenthal KM, Ellison CK, Tat CA, Walker HJ, Hastings PA, Burton RS. Establishing a DNA Sequence Database for the Marine Fish Fauna of California. [Unpublished and not examined during this study] May be the same specimen as MFC139-08.

GBGC6618-09: GenBank EU366564:

Davis MP. Evolutionary relationships of the Aulopiformes (Euteleostei: Cyclosquamata): a molecular and total evidence approach. In: Nelson JS, Schultze H-P, Wilson MVH. (Eds.) ORIGIN AND PHYLOGENETIC INTERRELATIONSHIPS OF TELEOSTS. Verlag Dr. Friedrich Pfeil, Munchen, Germany (in press 2010). [cited source not examined during this study]

K)

Origins of Specimens:

SCAFB: This study

FOAG: Australian Antarctic Territory (BOLD records)

GLF: Off Greenland (BOLD record)

MAECO: Mid-Atlantic Ridge (BOLD record)

MFC: Off California (BOLD record)

TZFPB: Off British Columbia (BOLD record)

GBGC6610-09: GenBank EU366573:

GBGC6611-09: GenBank EU366572:

Davis MP. Evolutionary relationships of the Aulopiformes (Euteleostei: Cyclosquamata): a molecular and total evidence approach. In: Nelson JS, Schultze H-P, Wilson MVH. (Eds.) ORIGIN AND PHYLOGENETIC INTERRELATIONSHIPS OF TELEOSTS. Verlag Dr. Friedrich Pfeil, Munchen, Germany (in press 2010). [cited source not examined during this study]

GenBank records the voucher specimen for both sequences as MCZ 158749. MCZ catalogue gives the capture location as in the vicinity of Bear Seamount.

L)

Origins of Specimens:

SCAFB, SCFAD: This study

CMNAF: David Strait (BOLD record)

DSFIB: Off Svalbard (BOLD record)

GLF: Off Greenland (BOLD record)

MAECO: Mid-Atlantic Ridge (BOLD record)

ANGBF1297-12: GenBank HQ167646:

Keskin E. Phylogenetic Relationship Among Myctophidae Species [Unpublished and not examined during this study].

GBGCA5860-13: GenBank AP012264:

Poulsen JY, Byrkjedal I, Willassen E, Rees D, Takeshima H, Satoh TP, Shinohara G, Nishida M, Miya M. Mitogenomic sequences and evidence from unique gene rearrangements corroborate evolutionary relationships of myctophiformes (Neoteleostei). BMC Evol Biol. 2013;13: 111.

Voucher specimen given as ZMUC P2393965. Sequence taken from the same specimen as GLF025 (Poulsen, *pers.comm.*).

GBGCA5280-13: GenBank KC616369:

Bernal A, Viñas J, Olivar MP. Genetic support for the morphological identification of larvae of Myctophidae, Gonostomadidae, Sternoptychidae and Phosichthyidae (Pisces) from the western Mediterranean. Scientia Marina. 2014;78(4).

Specimen caught near Balearics.

GBGCA5282-13: GenBank KC616367:

Bernal A, Viñas J, Olivar MP. Genetic support for the morphological identification of larvae of Myctophidae, Gonostomadidae, Sternoptychidae and Phosichthyidae (Pisces) from the western Mediterranean. Scientia Marina. 2014;78(4).

Specimen caught near Balearics.

GBGCA5283-13: GenBank KC616366:

Bernal A, Viñas J, Olivar MP. Genetic support for the morphological identification of larvae of Myctophidae, Gonostomadidae, Sternoptychidae and Phosichthyidae (Pisces) from the western Mediterranean. Scientia Marina. 2014;78(4).

Specimen caught near Balearics.

Sequence SBDSC006 had disappeared from BOLD by May 2017. The SBDSC project comprises sequences from benthic invertebrates taken off Newfoundland.

M)

Origins of Specimens:

SCAFB: This study

FPFLB: French Polynesia (BOLD record)

TZSAL: Off South Africa (BOLD record)

GBGCA11044-15: GenBank KJ555342:

Denton JS. Seven-locus molecular phylogeny of Myctophiformes (Teleostei; Scopelomorpha) highlights the utility of the order for studies of deep-sea evolution. Mol Phylogenet Evol. 2014; 76: 270-292.

In that paper, Table 1 gives the voucher specimen as AMNH 261119. The AMNH catalogue records that specimen as taken near American Samoa, at 14°49'S, 170°49'W.

GBGCA11043-15: GenBank KJ555341:

Denton JS. Seven-locus molecular phylogeny of Myctophiformes (Teleostei; Scopelomorpha) highlights the utility of the order for studies of deep-sea evolution. Mol Phylogenet Evol. 2014; 76: 270-292.

In that paper, Table 1 gives the voucher specimen as AMNH 261148. The AMNH catalogue records that specimen as taken near American Samoa, specifically “northwest of Tutuila”.

ANGBF8092: GenBank GU071724:

Bucklin A, Ortman BD, Jennings RM, Nigro LM, Sweetman CJ, Copley NJ, Sutton T, Wiebe PH. A 'Rosetta Stone' for metazoan zooplankton: DNA barcode analysis of species diversity of the Sargasso Sea (Northwest Atlantic Ocean). Deep Sea Res Part II. 2010;57: 2234-2247.

That paper’s Table 2 gives the capture location as 29.8°N 70.2°W.

N)

Origins of Specimens:

SCAFB: This study

MAECO: Mid-Atlantic Ridge (BOLD record)

GBGCA345-10: GenBank FJ918945:

DeVaney SC. Interrelationships of fishes of the order Stomiiformes. PhD Thesis.

Specimen caught over Bear Seamount (DeVaney, *pers.comm*.).

GBGCA5271: GenBank KC616378:

Bernal A, Viñas J, Olivar MP. Genetic support for the morphological identification of larvae of Myctophidae, Gonostomadidae, Sternoptychidae and Phosichthyidae (Pisces) from the western Mediterranean. Scientia Marina. 2014;78(4).

Specimen caught near Balearics.

GBGCA5272: GenBank KC616377:

Bernal A, Viñas J, Olivar MP. Genetic support for the morphological identification of larvae of Myctophidae, Gonostomadidae, Sternoptychidae and Phosichthyidae (Pisces) from the western Mediterranean. Scientia Marina. 2014;78(4).

Specimen caught near Balearics.

GBGCA5273: GenBank KC616376:

Bernal A, Viñas J, Olivar MP. Genetic support for the morphological identification of larvae of Myctophidae, Gonostomadidae, Sternoptychidae and Phosichthyidae (Pisces) from the western Mediterranean. Scientia Marina. 2014;78(4).

Specimen caught near Balearics.

O)

Origins of Specimens:

SCAFB: This study

DSSA: Mid-Atlantic Ridge in south Atlantic (BOLD record)

MAECO: Mid-Atlantic Ridge (BOLD record)

ANGBF8088: GenBank GU071732:

Bucklin A, Ortman BD, Jennings RM, Nigro LM, Sweetman CJ, Copley NJ, Sutton T, Wiebe PH. A 'Rosetta Stone' for metazoan zooplankton: DNA barcode analysis of species diversity of the Sargasso Sea (Northwest Atlantic Ocean). Deep Sea Res Part II. 2010;57:2234-2247.

That paper’s Table 2 gives the capture location as 24.9°N 60.5°W.

P)

Origins of Specimens:

SCAFB: This study

DSSAF: Mid-Atlantic Ridge in south Atlantic (BOLD record)

MAECO: Mid-Atlantic Ridge (BOLD record)

MFLI: Off Belize (BOLD record)

ANGBF369-12: GenBank GU071739:

Bucklin A, Ortman BD, Jennings RM, Nigro LM, Sweetman CJ, Copley NJ, Sutton T, Wiebe PH. A 'Rosetta Stone' for metazoan zooplankton: DNA barcode analysis of species diversity of the Sargasso Sea (Northwest Atlantic Ocean). Deep Sea Res Part II. 2010;57:2234-2247.

That paper’s Table 2 gives the capture location as 14.0°N 55.0°W.

Q)

Origins of Specimens:

SCAFB, SCFAC: This study

DSSAF: Mid-Atlantic Ridge in south Atlantic (BOLD record)

GLF: Off Greenland (BOLD record)

MAECO: Mid-Atlantic Ridge (BOLD record)

R)

Origins of Specimens:

SCAFB, SCFAC, SCFAD: This study

GLF: Off Greenland (BOLD record)

MAECO: Mid-Atlantic Ridge (BOLD record)

GBGCA5240: GenBank KC616409:

Bernal A, Viñas J, Olivar MP. Genetic support for the morphological identification of larvae of Myctophidae, Gonostomadidae, Sternoptychidae and Phosichthyidae (Pisces) from the western Mediterranean. Scientia Marine. 2014;78(4).

Specimen caught near Balearics.

GBGCA5861: GenBank AP012262:

Poulsen JY, Byrkjedal I, Willassen E, Rees D, Takeshima H, Satoh TP, Shinohara G, Nishida M, Miya M. Mitogenomic sequences and evidence from unique gene rearrangements corroborate evolutionary relationships of myctophiformes (Neoteleostei). BMC Evol. Biol. 2013;13, 111.

Voucher specimen given as ZMUC P2393970. The Zoological Museum of the University of Copenhagen (ZMUC) does not offer an up-to-date listing of its fish specimens on-line.

This sequence was developed from the same specimen as GLF030.S)

Origins of Specimens:

SCAFB: This study

FMV: Off Oregon (BOLD record)

MAECO: Mid-Atlantic Ridge (BOLD record)

MFC: Off California (BOLD record)

T)

Origins of Specimens:

SCAFB, SCFAC: This study

MAECO: Mid-Atlantic Ridge (BOLD record)

MFC: Off California (BOLD record)

CYTC4057-12: GenBank AB282850:

GBGCA578-10: GenBank NC013880:

Miya M, Pietsch TW, Orr JW, Arnold RJ, Satoh TP, Shedlock AM, Ho HC, Shimazaki M, Yabe M, Nishida M. Evolutionary history of anglerfishes (Teleostei: Lophiiformes): a mitogenomic perspective. BMC Evol Biol. 2010;10:58.

GenBank notes that the two sequences (AB282850 and NC013880) are identical but does not confirm that they are from the same specimen.

Sequence AB282850 came from a specimen taken off Shikoku Island, central Japan (Miya, *pers. comm.*).

GBGC4132-08: GenBank EU403054:

Gruenthal KM, Ellison CK, Tat CA, Walker HJ, Hastings PA, Burton RS. Establishing a DNA Sequence Database for the Marine Fish Fauna of California. [Unpublished and not examined during this study]

The latter sequence may be from the same specimen as MFC021-08.

U)

Origins of Specimens:

SCAFB: This study. Two specimens of the species were taken by the same set and a tissue sample for barcoding was taken from each.

MAECO: Mid-Atlantic Ridge (BOLD record)

GBGC0437-06: GenBank AP004423:

GBGC1592-06: GenBank NC004389 (derived from AP004423)

Miya M, Takeshima H, Endo H, Ishiguro NB, Inoue JG, Mukai T, Satoh TP, Yamaguchi M, Kawaguchi A, Mabuchi K, Shirai SM, Nishida M. Major patterns of higher teleostean phylogenies: a new perspective based on 100 complete mitochondrial DNA sequences. Mol Phylogenet Evol. 2003;26:121-138.

Specimen taken off Shikoku Island, central Japan (Dr. M.Miya, *pers.comm.*).

GBGC6286-09: GenBank AP010886:

GBGC8533-09: GenBank NC012044 (derived from AP010886)

Johnson GD, Paxton JR, Sutton TT, Satoh TP, Sado T, Nishida M, MiyaM. Deep-sea mystery solved: astonishing larval transformations and extreme sexual dimorphism unite three fish families. Biol Lett. 2009;5:235-239.

That paper’s Supplementary Table S1 identifies the voucher specimen as USNM391563. The USNM catalog gives the capture location as Strait of Florida, 24.2°N 79.31°W.

ANGBF383-12: GenBank GU071727:

Bucklin A, Ortman BD, Jennings RM, Nigro LM, Sweetman CJ, Copley NJ, Sutton T, Wiebe PH. A 'Rosetta Stone' for metazoan zooplankton: DNA barcode analysis of species diversity of the Sargasso Sea (Northwest Atlantic Ocean). Deep Sea Res Part II. 2010;57:2234-2247.

That paper’s Table 2 gives the capture location as 25.0°N 59.9°W.

GBGC6290-06: GenBank AP010882:

Johnson GD, Paxton JR, Sutton TT, Satoh TP, Sado T, Nishida M, MiyaM. Deep-sea mystery solved: astonishing larval transformations and extreme sexual dimorphism unite three fish families. Biol Lett. 2009;5:235-239.

That paper’s Supplementary Table S1 identifies the voucher specimen as MCZ164250. The MCZ catalog gives the capture location as Bear Seamount, 39°51'N 67°27'W.

V)

Origins of Specimens:

SCAFB: This study

FMV: Off Oregon (BOLD record)

MAECO: Mid-Atlantic Ridge (BOLD record)

MFC: Off California (BOLD record)

TZFPB: Off British Columbia (BOLD record)

W)

Origins of Specimens:

SCAFB: This study

CFSAN: Off Alabama (BOLD record)

DSLA: Off South Africa (BOLD record)

MFSP: Off Brazil (BOLD record)

X)

Origins of Specimens:

SCAFB, SCFAC, SCFAD: This study

FMV: Off Oregon (BOLD record)

MAECO: Mid-Atlantic Ridge (BOLD record)
