# Supplementary material for: Barcoding Atlantic Canada’s mesopelagic and upper bathypelagic marine fishes
Source: PLoS One. 2017 Sep 20;12(9):e0185173. doi: 10.1371/journal.pone.0185173 (PMC5607201; doi:10.1371/journal.pone.0185173)
Supplement: S1 Table — (DOCX) [file pone.0185173.s003.docx]

**S1 Table. List of Sequences in the Atlantic Canada Mesopelagic and Upper Bathypelagic Marine Fishes BOLD Projects ACMB and ACMF.**

| **Order, Family and Species** | **BOLD BIN** | **Field Sample Code** | **Voucher Specimen Catalogue Number** | **Process Reference Code** | **GenBank Accession Number**  **CO1-5P** |
| --- | --- | --- | --- | --- | --- |
| **Atlantic Canada Mesopelagic and Upper Bathypelagic Fishes of the Gully MPA (ACMB)** | | | | | |
| **ANGUILLIFORMES** |  |  |  |  |  |
| **Synaphobranchidae** |  |  |  |  |  |
| *Simenchelys parasitica* | BOLD:AAC0424 | 07-701 | ARC28373 | SCAFB1180-09 | KY033763 |
| *Synaphobranchus kaupii* | BOLD:AAA8286 | 08-108 | ARC28231 | SCAFB1303-09 | KY033770 |
| *Synaphobranchus kaupii* | BOLD:AAA8286 | 07-703 | ARC28659 | SCAFB1181-09 | KY033771 |
| *Synaphobranchus kaupii* | BOLD:AAA8286 | 07-650 | ARC28570 | SCAFB1147-09 | KY033772 |
| **Congridae** |  |  |  |  |  |
| *Xenomystax congroides* | BOLD:AAG4486 | 08-127 | ARC28261 | SCAFB1320-09 | KY033784 |
| **Derichthyidae** |  |  |  |  |  |
| *Derichthys serpentinus* | BOLD:AAC4336 | 07-612 | ARC28639 | SCAFB1119-09 | KY033607 |
| *Derichthys serpentinus* | BOLD:AAC4336 | 07-677 | ARC28569 | SCAFB1165-09 | KY033608 |
| *Derichthys serpentinus* | BOLD:AAC4336 | 08-053 | ARC28188 | SCAFB1250-09 | KY033609 |
| *Nessorhamphus ingolfianus* | BOLD:AAC8414 | 08-112.b | ARC28212 | SCAFB1307-09 | KY033700 |
| **Nemichthyidae** |  |  |  |  |  |
| *Avocettina infans* | BOLD:AAC9223 | 08-115 | ARC28207 | SCAFB1310-09 | KY033559 |
| *Nemichthys scolopaceus* | BOLD:AAB5787 | 08-022 | ARC28223 | SCAFB1220-09 | KY033698 |
| *Nemichthys scolopaceus* | BOLD:AAB5787 | 07-706 | ARC28621 | SCAFB1184-09 | KY033699 |
| **Serrivomeridae** |  |  |  |  |  |
| *Serrivomer beanii* | BOLD:AAB5786 | 08-020 | ARC28210 | SCAFB1218-09 | KY033755 |
| *Serrivomer lanceolatoides* | BOLD:AAC4861 | 07-656 | ARC28612 | SCAFB1151-09 | KY033756 |
| *Serrivomer lanceolatoides* | BOLD:AAC4861 | 07-645 | ARC28338 | SCAFB1143-09 | KY033757 |
| *Serrivomer lanceolatoides* | BOLD:AAC4861 | 08-008 | ARC28249 | SCAFB1207-09 | KY033758 |
| *Serrivomer lanceolatoides* | BOLD:AAC4861 | 07-603 | ARC28400 | SCAFB1113-09 | KY033759 |
| *Serrivomer lanceolatoides* | BOLD:AAC4861 | 08-095 | ARC28254 | SCAFB1292-09 | KY033760 |
| **Eurypharyngidae** |  |  |  |  |  |
| *Eurypharynx pelecanoides* | BOLD:AAB9456 | 08-107 | ARC28258 | SCAFB1302-09 | KY033615 |
| **ARGENTINIFORMES** |  |  |  |  |  |
| **Bathylagidae** |  |  |  |  |  |
| *Bathylagichthys greyae* | BOLD:AAD4198 | 07-540 | ARC28660 | SCFAD001-09 | KY033562 |
| *Bathylagichthys greyae* | BOLD:AAD4198 | 07-571 | ARC28376 | SCAFB1087-09 | KY033563 |
| *Bathylagichthys greyae* | BOLD:AAD4198 | 07-535 | ARC28632 | SCAFB1064-09 | KY033564 |
| *Bathylagichthys greyae* | BOLD:AAD4198 | 08-044 | ARC28206 | SCAFB1242-09 | KY033565 |
| *Bathylagichthys greyae* | BOLD:AAD4198 | 08-067 | ARC28178 | SCAFB1264-09 | KY033566 |
| *Bathylagus euryops* | BOLD:AAB5134 | 08-072 | ARC28230 | SCAFB1269-09 | KY033567 |
| *Bathylagus euryops* | BOLD:AAB5134 | 08-045 | ARC28241 | SCAFB1243-09 | KY033568 |
| *Bathylagus euryops* | BOLD:AAB5134 | 07-600 | ARC28649 | SCAFB1110-09 | KY033569 |
| *Bathylagus euryops* | BOLD:AAB5134 | 07-680 | ARC28607 | SCAFB1168-09 | KY033570 |
| *Dolicholagus longirostris* | BOLD:AAD4197 | 07-707 | ARC28409 | SCAFB1185-09 | KY033613 |
| *Dolicholagus longirostris* | BOLD:AAD4197 | 08-032 | ARC28239 | SCAFB1230-09 | KY033614 |
| **ALEPOCEPHALIFORMES** |  |  |  |  |  |
| **Alepocephalidae** |  |  |  |  |  |
| *Alepocephalus agassizii* | BOLD:AAD2224 | 07-695 | ARC28292 | SCAFB1175-09 | KY033543 |
| *Xenodermichthys copei* | BOLD:AAB2780 | 08-091 | ARC28217 | SCAFB1288-09 | KY033780 |
| *Xenodermichthys copei* | BOLD:AAB2780 | 07-613 | ARC28644 | SCAFB1120-09 | KY033781 |
| *Xenodermichthys copei* | BOLD:AAB2780 | 08-054 | ARC28192 | SCAFB1251-09 | KY033782 |
| **Platytroctidae** |  |  |  |  |  |
| *Barbantus curvifrons* | BOLD:AAI7630 | 08-098 | ARC28268 | SCAFB1295-09 | KY033560 |
| *Holtbyrnia anomala* | BOLD:AAC3092 | 07-659 | ARC28389 | SCAFB1154-09 | KY033631 |
| *Holtbyrnia anomala* | BOLD:AAC3092 | 08-061 | ARC28263 | SCAFB1258-09 | KY033632 |
| *Maulisia microlepis* | BOLD:AAD4188 | 07-584 | ARC28394 | SCAFB1097-09 | KY033668 |
| *Normichthys operosus* | BOLD:AAC3091 | 07-660 | ARC28389 | SCAFB1155-09 | KY033703 |
| *Normichthys operosus* | BOLD:AAC3091 | 07-653 | ARC28368 | SCAFB1150-09 | KY033702 |
| **STOMIIFORMES** |  |  |  |  |  |
| **Gonostomatidae** |  |  |  |  |  |
| *Cyclothone microdon* | BOLD:AAB4944 | 08-084 | ARC28278 | SCAFB1281-09 | KY033602 |
| *Cyclothone microdon* | BOLD:AAB4944 | 08-102 | ARC28170 | SCAFB1299-09 | KY033604 |
| *Cyclothone microdon* | BOLD:AAB4944 | 07-757 | ARC28559 | SCFAD050-09 | KY033603 |
| *Cyclothone pallida* | BOLD:AAC8495 | 07-647 | ARC28349 | SCAFB1145-09 | KY033605 |
| *Cyclothone pallida* | BOLD:AAC8495 | 07-605 | ARC28366 | SCAFB1115-09 | KY033606 |
| *Gonostoma atlanticum* | BOLD:AAD1425 | 07-557 | ARC28550 | SCAFB1079-09 | KY033623 |
| *Gonostoma atlanticum* | BOLD:AAD1425 | 08-080 | ARC28181 | SCAFB1277-09 | KY033624 |
| *Gonostoma elongatum* | BOLD:AAB2394 | 07-713 | ARC28405 | SCAFB1189-09 | KY033625 |
| *Gonostoma elongatum* | BOLD:AAB2394 | 08-031 | ARC28238 | SCAFB1229-09 | KY033626 |
| *Gonostoma elongatum* | BOLD:AAB2394 | 07-721 | ARC28288 | SCAFB1197-09 | KY033627 |
| *Sigmops bathyphilus* | BOLD:AAC7744 | 07-583 | ARC28362 | SCAFB1096-09 | KY033761 |
| *Sigmops bathyphilus* | BOLD:AAC7744 | 07-569 | ARC28362 | SCAFB1086-09 | KY033762 |
| **Sternoptychidae** |  |  |  |  |  |
| *Argyropelecus aculeatus* | BOLD:AAB6595 | 08-034 | ARC28216 | SCAFB1232-09 | KY033551 |
| *Argyropelecus aculeatus* | BOLD:AAB6595 | 07-526 | ARC28626 | SCAFB1055-09 | KY033552 |
| *Argyropelecus gigas* | BOLD:AAC0977 | 07-622 | ARC28382 | SCAFB1125-09 | KY033553 |
| *Argyropelecus gigas* | BOLD:AAC0977 | 07-609 | ARC28665 | SCAFB1117-09 | KY033554 |
| *Argyropelecus gigas* | BOLD:AAC0977 | 08-035 | ARC28240 | SCAFB1233-09 | KY033555 |
| *Argyropelecus gigas* | BOLD:AAC0977 | 07-608 | ARC28665 | SCAFB1116-09 | KY033556 |
| *Argyropelecus hemigymnus* | BOLD:AAC6584 | 07-726 | ARC28546 | SCFAD005-09 | KY033557 |
| *Argyropelecus hemigymnus* | BOLD:AAC6584 | 08-018 | ARC28199 | SCAFB1216-09 | KY033558 |
| *Maurolicus weitzmani* | BOLD:AAB8653 | 07-530 | ARC28545 | SCAFB1059-09 | KY033669 |
| *Maurolicus weitzmani* | BOLD:AAB8653 | 07-529 | ARC28545 | SCAFB1058-09 | KY033670 |
| *Polyipnus clarus* | BOLD:AAB6596 | 07-766A | ARC28599 | SCFAD058-09 | KY033731 |
| *Polyipnus clarus* | BOLD:AAB6596 | 07-640 | ARC28345 | SCAFB1138-09 | KY033732 |
| *Polyipnus clarus* | BOLD:AAB6596 | 08-068 | ARC28177 | SCAFB1265-09 | KY033733 |
| *Sternoptyx diaphana* | BOLD:AAI4421 | 08-023 | ARC28245 | SCAFB1221-09 | KY033764 |
| *Valenciennellus tripunctulatus* | BOLD:AAH7815 | 08-071 | ARC28179 | SCAFB1268-09 | KY033777 |
| **Phosichthyidae** |  |  |  |  |  |
| *Vinciguerria nimbaria* | BOLD:AAD3594 | 07-711 | ARC28548 | SCAFB1187-09 | KY033778 |
| *Vinciguerria nimbaria* | BOLD:AAD3594 | 08-004 | ARC28200 | SCAFB1203-09 | KY033779 |
| **Stomiidae** |  |  |  |  |  |
| *Bathophilus vaillanti* | BOLD:AAW2854 | 08-113 | ARC28221 | SCAFB1308-09 | KY033561 |
| *Borostomias antarcticus* | BOLD:AAB5140 | 07-696 | ARC28290 | SCAFB1176-09 | KY033575 |
| *Borostomias antarcticus* | BOLD:AAB5140 | 07-566 | ARC28370 | SCAFB1084-09 | KY033576 |
| *Borostomias antarcticus* | BOLD:AAB5140 | 08-066 | ARC28252 | SCAFB1263-09 | KY033577 |
| *Chauliodus sloani* | BOLD:AAB1178 | 08-005 | ARC28215 | SCAFB1204-09 | KY033589 |
| *Flagellostomias boureei* | BOLD:AAD8807 | 07-630 | ARC28381 | SCAFB1130-09 | KY033620 |
| *Flagellostomias boureei* | BOLD:AAD8807 | 08-121 | ARC28174 | SCAFB1315-09 | KY033621 |
| *Idiacanthus fasciola* | BOLD:AAK4190 | 08-056 | ARC28191 | SCAFB1253-09 | KY033637 |
| *Malacosteus niger* | BOLD:AAB5515 | 07-809 | ARC28377 | SCFAD504-09 | KY033665 |
| *Malacosteus niger* | BOLD:AAB5515 | 07-698 | ARC28641 | SCAFB1178-09 | KY033666 |
| *Malacosteus niger* | BOLD:AAB5515 | 08-057 | ARC28194 | SCAFB1254-09 | KY033667 |
| *Leptostomias gladiator* | BOLD:AAJ1507 | 08-101 | ARC28256 | SCAFB1298-09 | KY033656 |
| *Pachystomias microdon* | BOLD:AAD6660 | 07-598 | ARC28408 | SCAFB1108-09 | KY033721 |
| *Pachystomias microdon* | BOLD:AAD6660 | 07-591 | ARC28407 | SCAFB1103-09 | KY033722 |
| *Photostomias guernei* | BOLD:AAB5517 | 08-007 | ARC28220 | SCAFB1206-09 | KY033726 |
| *Photostomias guernei* | BOLD:AAB5517 | 08-058 | ARC28175 | SCAFB1255-09 | KY033727 |
| *Photostomias guernei* | BOLD:AAB5517 | 07-517 | ARC28655 | SCAFB1046-09 | KY033728 |
| *Photostomias guernei* | BOLD:AAB5517 | 07-520 | ARC28655 | SCAFB1049-09 | KY033729 |
| *Photostomias guernei* | BOLD:AAB5517 | 07-777 | ARC28625 | SCFAD072-09 | KY033730 |
| **AULOPIFORMES** |  |  |  |  |  |
| **Chlorophthalmidae** |  |  |  |  |  |
| *Chlorophthalmus agassizi* | BOLD:AAB2600 | 08-092 | ARC28166 | SCAFB1289-09 | KY033596 |
| *Chlorophthalmus agassizi* | BOLD:AAB2600 | 08-090 | ARC28165 | SCAFB1287-09 | KY033597 |
| *Chlorophthalmus agassizi* | BOLD:AAB2600 | 07-588 | ARC28341 | SCAFB1101-09 | KY033598 |
| **Notosudidae** |  |  |  |  |  |
| *Scopelosaurus lepidus* | BOLD:AAA8786 | 08-128 | ARC28164 | SCAFB1321-09 | KY033753 |
| *Scopelosaurus lepidus* | BOLD:AAA8786 | 07-554 | ARC28565 | SCAFB1076-09 | KY033754 |
| **Giganturidae** |  |  |  |  |  |
| *Gigantura chuni* | BOLD:AAG8668 | 07-658 | ARC28624 | SCAFB1153-09 | KY033622 |
| **Paralepididae** |  |  |  |  |  |
| *Arctozenus risso* | BOLD:AAA8780 | 07-523 | ARC28664 | SCAFB1052-09 | KY033547 |
| *Arctozenus risso* | BOLD:AAA8780 | 08-014 | ARC28243 | SCAFB1213-09 | KY033548 |
| *Arctozenus risso* | BOLD:AAA8780 | 07-522 | ARC28664 | SCAFB1051-09 | KY033549 |
| *Arctozenus risso* | BOLD:AAA8780 | 07-752 | ARC28620 | SCFAD042-09 | KY033550 |
| *Magnisudis atlantica* | BOLD:AAB9276 | 08-077 | ARC28208 | SCAFB1274-09 | KY033663 |
| *Magnisudis atlantica* | BOLD:AAB9276 | 08-055 | ARC28193 | SCAFB1252-09 | KY033664 |
| **Evermannellidae** |  |  |  |  |  |
| *Evermannella balbo* | BOLD:AAD1201 | 08-059 | ARC28251 | SCAFB1256-09 | KY033616 |
| *Evermannella balbo* | BOLD:AAD1201 | 07-516 | ARC28399 | SCAFB1045-09 | KY033617 |
| *Evermannella balbo* | BOLD:AAD1201 | 07-662 | ARC28380 | SCAFB1156-09 | KY033618 |
| *Evermannella balbo* | BOLD:AAD1201 | 07-561 | ARC28352 | SCAFB1081-09 | KY033619 |
| **Alepisauridae** |  |  |  |  |  |
| *Alepisaurus brevirostris* | BOLD:AAC5236 | 07-624 | ARC28634 | SCAFB1127-09 | KY033538 |
| *Alepisaurus ferox* | BOLD:AAC5235 | 07-501 | ARC28418 | SCAFB1030-09 | KY033541 |
| *Alepisaurus ferox* | BOLD:AAC5236 | 08-081 | ARC28205 | SCAFB1278-09 | KY033539 |
| *Alepisaurus ferox* | BOLD:AAC5236 | 07-681 | ARC28287 | SCAFB1169-09 | KY033540 |
| *Alepisaurus ferox* | BOLD:AAC5236 | 07-610 | ARC28291 | SCAFB1118-09 | KY033542 |
| *Omosudis lowii* | BOLD:AAD3023 | 07-679 | ARC28543 | SCAFB1167-09 | KY033716 |
| *Omosudis lowii* | BOLD:AAD3023 | 08-083 | ARC28219 | SCAFB1280-09 | KY033717 |
| *Omosudis lowii* | BOLD:AAD3023 | 08-039 | ARC28228 | SCAFB1237-09 | KY033718 |
| *Omosudis lowii* | BOLD:AAD3023 | 07-623 | ARC28374 | SCAFB1126-09 | KY033719 |
| **MYCTOPHIFORMES** |  |  |  |  |  |
| **Myctophidae** |  |  |  |  |  |
| *Benthosema glaciale* | BOLD:AAC5632 | 08-012 | ARC28242 | SCAFB1211-09 | KY033571 |
| *Benthosema glaciale* | BOLD:AAC5632 | 07-806 | ARC28583 | SCFAD093-09 | KY033572 |
| *Bolinichthys indicus* | BOLD:AAF6566 | 08-036 | ARC28186 | SCAFB1234-09 | KY033573 |
| *Bolinichthys photothorax* | BOLD:AAJ7869 | 08-026 | ARC28203 | SCAFB1224-09 | KY033574 |
| *Ceratoscopelus maderensis* | BOLD:AAC2875 | 07-533 | ARC28651 | SCAFB1062-09 | KY033579 |
| *Ceratoscopelus maderensis* | BOLD:AAC2875 | 08-052 | ARC28189 | SCAFB1249-09 | KY033580 |
| *Ceratoscopelus maderensis* | BOLD:AAC2875 | 07-534 | ARC28651 | SCAFB1063-09 | KY033581 |
| *Ceratoscopelus maderensis* | BOLD:AAC2875 | 08-015 | ARC28247 | SCAFB1214-09 | KY033582 |
| *Ceratoscopelus maderensis* | BOLD:AAC2875 | 07-646 | ARC28627 | SCAFB1144-09 | KY033583 |
| *Ceratoscopelus warmingii* | BOLD:AAC3131 | 08-120 | ARC28173 | SCAFB1314-09 | KY033584 |
| *Diaphus dumerilii* | BOLD:AAF5612 | 08-027 | ARC28202 | SCAFB1225-09 | KY033610 |
| *Diaphus mollis* | BOLD:AAC9267 | 08-096 | ARC28168 | SCAFB1293-09 | KY033611 |
| *Hygophum hygomii* | BOLD:AAC6495 | 07-504 | ARC28358 | SCAFB1033-09 | KY033634 |
| *Hygophum hygomii* | BOLD:AAC6495 | 08-019 | ARC28198 | SCAFB1217-09 | KY033635 |
| *Hygophum hygomii* | BOLD:AAC6495 | 07-505 | ARC28358 | SCAFB1034-09 | KY033636 |
| *Lampadena speculigera* | BOLD:AAC2331 | 07-736 | ARC28378 | SCFAD495-09 | KY033641 |
| *Lampadena speculigera* | BOLD:AAC2331 | 07-512 | ARC28404 | SCAFB1041-09 | KY033642 |
| *Lampadena speculigera* | BOLD:AAC2331 | 08-010 | ARC28236 | SCAFB1209-09 | KY033643 |
| *Lampadena speculigera* | BOLD:AAC2331 | 08-037 | ARC28236 | SCAFB1235-09 | KY033644 |
| *Lampadena speculigera* | BOLD:AAC2331 | 07-749 | ARC28392 | SCFAD496-09 | KY033645 |
| *Lampadena speculigera* | BOLD:AAC2331 | 07-513 | ARC28404 | SCAFB1042-09 | KY033646 |
| *Lampanyctus festivus* | BOLD:AAD5275 | 08-118 | ARC28172 | SCAFB1312-09 | KY033647 |
| *Lampanyctus macdonaldi* | BOLD:AAB3777 | 07-579 | ARC28363 | SCAFB1092-09 | KY033648 |
| *Lampanyctus macdonaldi* | BOLD:AAB3777 | 08-024 | ARC28222 | SCAFB1222-09 | KY033649 |
| *Lampanyctus macdonaldi* | BOLD:AAB3777 | 07-578 | ARC28363 | SCAFB1091-09 | KY033650 |
| *Lampanyctus photonotus* | BOLD:AAF2850 | 08-043 | ARC28187 | SCAFB1241-09 | KY033651 |
| *Lampanyctus pusillus* | BOLD:AAE2725 | 08-079 | ARC28182 | SCAFB1276-09 | KY033652 |
| *Lepidophanes guentheri* | BOLD:AAD1806 | 08-074 | ARC28180 | SCAFB1271-09 | KY033654 |
| *Lepidophanes guentheri* | BOLD:AAD1806 | 08-029 | ARC28204 | SCAFB1227-09 | KY033655 |
| *Lobianchia dofleini* | BOLD:AAC1960 | 07-502 | ARC28348 | SCAFB1031-09 | KY033657 |
| *Lobianchia dofleini* | BOLD:AAC1960 | 07-524 | ARC28344 | SCAFB1053-09 | KY033658 |
| *Lobianchia dofleini* | BOLD:AAC1960 | 08-013 | ARC28195 | SCAFB1212-09 | KY033659 |
| *Lobianchia dofleini* | BOLD:AAC1960 | 07-525 | ARC28344 | SCAFB1054-09 | KY033660 |
| *Myctophum affine* | BOLD:AAD9568 | 07-717 | ARC28359 | SCAFB1193-09 | KY033679 |
| *Myctophum affine* | BOLD:AAD9568 | 08-093 | ARC28167 | SCAFB1290-09 | KY033680 |
| *Myctophum affine* | BOLD:AAD9568 | 08-009 | ARC28196 | SCAFB1208-09 | KY033681 |
| *Myctophum punctatum* | BOLD:AAB5848 | 07-750A | ARC28604 | SCFAD039-09 | KY033682 |
| *Myctophum punctatum* | BOLD:AAB5848 | 07-769C | ARC28653 | SCFAD063-09 | KY033683 |
| *Myctophum punctatum* | BOLD:AAB5848 | 08-011 | ARC28244 | SCAFB1210-09 | KY033684 |
| *Myctophum punctatum* | BOLD:AAB5848 | 08-106 | ARC28171 | SCAFB1301-09 | KY033685 |
| *Myctophum punctatum* | BOLD:AAB5848 | 07-643 | ARC28383 | SCAFB1141-09 | KY033686 |
| *Myctophum punctatum* | BOLD:AAB5848 | 07-750B | ARC28604 | SCFAD040-09 | KY033687 |
| *Myctophum punctatum* | BOLD:AAB5848 | 07-644 | ARC28383 | SCAFB1142-09 | KY033688 |
| *Nannobrachium atrum* | BOLD:AAB3779 | 07-576 | ARC28415 | SCAFB1089-09 | KY033689 |
| *Nannobrachium atrum* | BOLD:AAB3779 | 08-006 | ARC28201 | SCAFB1205-09 | KY033690 |
| *Nannobrachium atrum* | BOLD:AAB3779 | 07-580 | ARC28595 | SCAFB1093-09 | KY033691 |
| *Nannobrachium atrum* | BOLD:AAB3779 | 07-581 | ARC28595 | SCAFB1094-09 | KY033692 |
| *Nannobrachium lineatum* | BOLD:AAB5303 | 07-577 | ARC28415 | SCAFB1090-09 | KY033693 |
| *Notoscopelus bolini* | BOLD:AAC4628 | 07-531 | ARC28364 | SCAFB1060-09 | KY033704 |
| *Notoscopelus bolini* | BOLD:AAC4628 | 08-003 | ARC28237 | SCAFB1202-09 | KY033705 |
| *Notoscopelus bolini* | BOLD:AAC4628 | 07-582 | ARC28648 | SCAFB1095-09 | KY033706 |
| *Notoscopelus bolini* | BOLD:AAC4628 | 07-532 | ARC28364 | SCAFB1061-09 | KY033707 |
| *Notoscopelus elongatus* | BOLD:AAB7073 | 07-620 | ARC28347 | SCAFB1124-09 | KY033708 |
| *Notoscopelus elongatus* | BOLD:AAB7073 | 07-771B | ARC28596 | SCFAD067-09 | KY033709 |
| *Notoscopelus elongatus* | BOLD:AAB7073 | 07-771C | ARC28596 | SCFAD068-09 | KY033710 |
| *Notoscopelus elongatus* | BOLD:AAB7073 | 08-042 | ARC28280 | SCAFB1240-09 | KY033711 |
| *Notoscopelus resplendens* | BOLD:AAD4302 | 07-506 | ARC28350 | SCAFB1035-09 | KY033712 |
| *Notoscopelus resplendens* | BOLD:AAD4302 | 08-017 | ARC28246 | SCAFB1215-09 | KY033713 |
| *Notoscopelus resplendens* | BOLD:AAD4302 | 08-002 | ARC28248 | SCAFB1201-09 | KY033714 |
| *Notoscopelus resplendens* | BOLD:AAD4302 | 07-507 | ARC28354 | SCAFB1036-09 | KY033715 |
| *Protomyctophum arcticum* | BOLD:ACR2195 | 08-030 | ARC28185 | SCAFB1228-09 | KY033741 |
| *Protomyctophum arcticum* | BOLD:ACR2195 | 07-587 | ARC28346 | SCAFB1100-09 | KY033742 |
| *Protomyctophum arcticum* | BOLD:ACR2195 | 07-586 | ARC28361 | SCAFB1099-09 | KY033743 |
| *Symbolophorus veranyi* | BOLD:AAC4870 | 07-553 | ARC28597 | SCAFB1075-09 | KY033765 |
| *Symbolophorus veranyi* | BOLD:AAC4870 | 07-552 | ARC28597 | SCAFB1074-09 | KY033766 |
| *Symbolophorus veranyi* | BOLD:AAC4870 | 07-744C | ARC28658 | SCFAD033-09 | KY033767 |
| *Symbolophorus veranyi* | BOLD:AAC4870 | 07-537 | ARC28663 | SCAFB1066-09 | KY033768 |
| *Symbolophorus veranyi* | BOLD:AAC4870 | 07-536 | ARC28663 | SCAFB1065-09 | KY033769 |
| *Taaningichthys bathyphilus* | BOLD:AAC2930 | 07-665 | ARC28343 | SCAFB1158-09 | KY033773 |
| *Taaningichthys bathyphilus* | BOLD:AAC2930 | 07-627 | ARC28342 | SCAFB1129-09 | KY033774 |
| *Taaningichthys bathyphilus* | BOLD:AAC2930 | 07-668 | ARC28353 | SCAFB1161-09 | KY033775 |
| *Taaningichthys bathyphilus* | BOLD:AAC2930 | 08-099 | ARC28169 | SCAFB1296-09 | KY033776 |
| **GADIFORMES** |  |  |  |  |  |
| **Macrouridae** |  |  |  |  |  |
| *Nezumia bairdii* | BOLD:AAB9334 | 07-705 | ARC28369 | SCAFB1183-09 | KY033701 |
| **Moridae** |  |  |  |  |  |
| *Laemonema barbatulum* | BOLD:AAF2623 | 08-130 | ARC28286 | SCAFB1323-09 | KY033639 |
| *Laemonema barbatulum* | BOLD:AAF2623 | 07-631 | ARC28386 | SCAFB1131-09 | KY033640 |
| *Laemonema barbatulum* | BOLD:AAF2623 | 07-708 | ARC28385 | SCAFB1186-09 | KY033638 |
| **Melanonidae** |  |  |  |  |  |
| *Melanonus zugmayeri* | BOLD:AAC7280 | 07-594 | ARC28585 | SCAFB1104-09 | KY033677 |
| **OPHIDIIFORMES** |  |  |  |  |  |
| **Ophidiidae** |  |  |  |  |  |
| *Lamprogrammus brunswigi* | BOLD:AAI3287 | 07-694 | ARC28413 | SCAFB1174-09 | KY033653 |
| **LOPHIIFORMES** |  |  |  |  |  |
| **Melanocetidae** |  |  |  |  |  |
| *Melanocetus johnsonii* | BOLD:AAC8494 | 08-122 | ARC28273 | SCAFB1316-09 | KY033676 |
| **Oneirodidae** |  |  |  |  |  |
| *Chaenophryne longiceps* | BOLD:AAD9322 | 08-094 | ARC28265 | SCAFB1291-09 | KY033587 |
| *Lophodolos acanthognathus* | BOLD:AAH3566 | 07-720b | ARC28395 | SCFAD493-09 | KY033661 |
| *Lophodolos acanthognathus* | BOLD:AAH3566 | 07-720a | ARC28395 | SCFAD492-09 | KY033662 |
| *Oneirodes bradburyae* | BOLD:AAD9323 | 07-686 | ARC28615 | SCAFB1172-09 | KY033720 |
| **Ceratiidae** |  |  |  |  |  |
| *Ceratias holboelli* | BOLD:AAC1510 | 08-088 | ARC28214 | SCAFB1285-09 | KY033578 |
| *Cryptopsaras couesii* | BOLD:AAC1509 | 07-617 | ARC28227 | SCAFB1121-09 | KY033599 |
| *Cryptopsaras couesii* | BOLD:AAC1509 | 07-596 | ARC28610 | SCAFB1106-09 | KY033600 |
| *Cryptopsaras couesii* | BOLD:AAC1509 | 08-060 | ARC28209 | SCAFB1257-09 | KY033601 |
| **Linophyrnidae** |  |  |  |  |  |
| *Haplophryne mollis* | BOLD:AAF1819 | 08-129 | ARC28262 | SCAFB1322-09 | KY033628 |
| *Haplophryne mollis* | BOLD:AAF1819 | 07-604 | ARC28384 | SCAFB1114-09 | KY033629 |
| *Haplophryne mollis* | BOLD:AAF1819 | 07-597 | ARC28661 | SCAFB1107-09 | KY033630 |
| **BERYCIFORMES** |  |  |  |  |  |
| **Melamphaidae** |  |  |  |  |  |
| *Melamphaes suborbitalis* | BOLD:AAC4479 | 07-590 | ARC28411 | SCAFB1102-09 | KY033671 |
| *Melamphaes suborbitalis* | BOLD:AAC4479 | 07-555 | ARC28563 | SCAFB1077-09 | KY033672 |
| *Melamphaes suborbitalis* | BOLD:AAC4479 | 08-062 | ARC28218 | SCAFB1259-09 | KY033673 |
| *Melamphaes suborbitalis* | BOLD:AAC4479 | 08-041 | ARC28235 | SCAFB1239-09 | KY033674 |
| *Melamphaes suborbitalis* | BOLD:AAC4479 | 07-556 | ARC28563 | SCAFB1078-09 | KY033675 |
| *Poromitra capito* | BOLD:AAC5610 | 08-048 | ARC28190 | SCAFB1245-09 | KY033734 |
| *Poromitra crassiceps* | BOLD:AAB7775 | 07-716 | ARC28371 | SCAFB1192-09 | KY033735 |
| *Poromitra crassiceps* | BOLD:AAB7775 | 07-684 | ARC28567 | SCAFB1171-09 | KY033736 |
| *Poromitra megalops* | BOLD:AAC7683 | 08-085 | ARC28279 | SCAFB1282-09 | KY033737 |
| *Poromitra megalops* | BOLD:AAC7683 | 07-719 | ARC28564 | SCAFB1195-09 | KY033738 |
| *Poromitra megalops* | BOLD:AAC7683 | 08-040 | ARC28281 | SCAFB1238-09 | KY033739 |
| *Scopeloberyx opisthopterus* | BOLD:AAC4478 | 08-050 | ARC28282 | SCAFB1247-09 | KY033748 |
| *Scopeloberyx opisthopterus* | BOLD:AAC4478 | 08-063 | ARC28283 | SCAFB1260-09 | KY033749 |
| *Scopeloberyx opisthopterus* | BOLD:AAC4478 | 07-741B | ARC28572 | SCFAD026-09 | KY033750 |
| *Scopelogadus beanii* | BOLD:AAB8806 | 07-514 | ARC28403 | SCAFB1043-09 | KY033751 |
| *Scopelogadus mizolepis* | BOLD:AAB8806 | 07-515 | ARC28403 | SCAFB1044-09 | KY033752 |
| **Rondeletiidae** |  |  |  |  |  |
| *Rondeletia loricata* | BOLD:AAB8446 | 07-521 | ARC28631 | SCAFB1050-09 | KY033745 |
| *Rondeletia loricata* | BOLD:AAB8446 | 08-109 | ARC28259 | SCAFB1304-09 | KY033746 |
| *Rondeletia loricata* | BOLD:AAB8446 | 08-087 | ARC28253 | SCAFB1284-09 | KY033747 |
| **Cetomimidae** |  |  |  |  |  |
| *Cetostoma regani* | BOLD:AAD4255 | 07-509 | ARC28401 | SCAFB1038-09 | KY033585 |
| *Cetostoma regani* | BOLD:AAD4255 | 07-508 | ARC28401 | SCAFB1037-09 | KY033586 |
| **TRACHICHTHYIFORMES** |  |  |  |  |  |
| **Anoplogastridae** |  |  |  |  |  |
| *Anoplogaster cornuta* | BOLD:AAB6136 | 07-813 | ARC28614 | SCFAD100-09 | KY033544 |
| *Anoplogaster cornuta* | BOLD:AAB6136 | 08-033 | ARC28211 | SCAFB1231-09 | KY033545 |
| *Anoplogaster cornuta* | BOLD:AAB6136 | 07-712 | ARC28340 | SCAFB1188-09 | KY033546 |
| **ZEIFORMES** |  |  |  |  |  |
| **Grammicolepididae** |  |  |  |  |  |
| *Xenolepidichthys dalgleishi* | BOLD:AAC6356 | 08-116 | ARC28260 | SCAFB1311-09 | KY033783 |
| **SCORPAENIFORMES** |  |  |  |  |  |
| **Zoarcidae** |  |  |  |  |  |
| *Melanostigma atlanticum* | BOLD:AAC0509 | 08-089 | ARC28184 | SCAFB1286-09 | KY033678 |
| **Liparidae** |  |  |  |  |  |
| *Paraliparis copei* | BOLD:ABZ4378 | 07-639 | ARC28379 | SCAFB1137-09 | KY033723 |
| *Paraliparis copei* | BOLD:ABZ4378 | 08-103 | ARC28257 | SCAFB1300-09 | KY033724 |
| *Paraliparis copei* | BOLD:ABZ4378 | 07-638 | ARC28379 | SCAFB1136-09 | KY033725 |
| **PERCIFORMES** |  |  |  |  |  |
| **Howellidae** |  |  |  |  |  |
| *Howella brodiei* | BOLD:AAE1485 | 08-086 | ARC28183 | SCAFB1283-09 | KY033633 |
| **Priacanthidae** |  |  |  |  |  |
| *Priacanthus arenatus* | BOLD:ABZ5909 | 07-663 | ARC28571 | SCAFB1157-09 | KY033740 |
| **Chaetodontidae** |  |  |  |  |  |
| *Chaetodon ocellatus* | BOLD:AAC0425 | 07-652 | ARC28579 | SCAFB1149-09 | KY033588 |
| **SCOMBRIFORMES** |  |  |  |  |  |
| **Gempylidae** |  |  |  |  |  |
| *Diplospinus multistriatus* | BOLD:AAD7109 | 08-125 | ARC28224 | SCAFB1318-09 | KY033612 |
| *Nealotus tripes* | BOLD:AAC6290 | 08-038 | ARC28232 | SCAFB1236-09 | KY033694 |
| *Nealotus tripes* | BOLD:AAC6290 | 08-100 | ARC28272 | SCAFB1297-09 | KY033696 |
| *Nealotus tripes* | BOLD:AAC6290 | 07-519 | ARC28398 | SCAFB1048-09 | KY033697 |
| *Nealotus tripes* | BOLD:ADB0794 | 08-021 | ARC28232 | SCAFB1219-09 | KY033695 |
| **TRACHINIFORMES** |  |  |  |  |  |
| **Chiasmodontidae** |  |  |  |  |  |
| *Chiasmodon niger* | BOLD:AAB4943 | 07-602 | ARC28630 | SCAFB1112-09 | KY033590 |
| *Chiasmodon niger* | BOLD:AAB4943 | 07-601 | ARC28630 | SCAFB1111-09 | KY033591 |
| *Chiasmodon niger* | BOLD:AAB4943 | 07-618 | ARC28393 | SCAFB1122-09 | KY033592 |
| *Chiasmodon niger* | BOLD:AAB4943 | 07-748 | ARC28587 | SCFAD038-09 | KY033593 |
| *Chiasmodon niger* | BOLD:AAB4943 | 07-678 | ARC28339 | SCAFB1166-09 | KY033594 |
| *Chiasmodon niger* | BOLD:AAB4943 | 07-672 | ARC28635 | SCAFB1162-09 | KY033595 |
| *Pseudoscopelus astronesthidens* | BOLD:AAF6631 | 08-069 | ARC28269 | SCAFB1266-09 | KY033744 |
|  |  |  |  |  |  |
| **Atlantic Canada Mesopelagic Fishes (ACMF)** | | | | | |
| **ANGUILLIFORMES** |  |  |  |  |  |
| **Synaphobranchidae** |  |  |  |  |  |
| *Simenchelys parasitica* | BOLD:AAC0424 | 06-732 | ARC26755 | SCFAC654-06 | KY033947 |
| *Simenchelys parasitica* | BOLD:AAC0424 | 07-394 | ARC26895 | SCAFB1006-07 | KY033948 |
| *Synaphobranchus kaupii* | BOLD:AAA8286 | 09-378 | ARC28321 | SCFAD460-09 | KY033960 |
| *Synaphobranchus kaupii* | BOLD:AAA8286 | 07-224 | ARC26821 | SCAFB836-07 | KY033961 |
| *Synaphobranchus kaupii* | BOLD:AAA8286 | 06-693 | ARC26177 | SCFAC785-06 | KY033962 |
| *Synaphobranchus kaupii* | BOLD:AAA8286 | 07-223 | ARC26821 | SCAFB835-07 | KY033963 |
| **Nemichthyidae** |  |  |  |  |  |
| *Nemichthys scolopaceus* | BOLD:AAB5787 | 07-229 | ARC26826 | SCAFB841-07 | KY033920 |
| **Serrivomeridae** |  |  |  |  |  |
| *Serrivomer beanii* | BOLD:AAB5786 | 96-016 | ARC25867 | SCFAC887-06 | KY033944 |
| *Serrivomer beanii* | BOLD:AAB5786 | 96-007 | ARC25836 | SCFAC669-06 | KY033945 |
| *Serrivomer beanii* | BOLD:AAB5786 | 06-011 | ARC25682 | SCFAC315-06 | KY033946 |
| **Eurypharyngidae** |  |  |  |  |  |
| *Eurypharynx pelecanoides* | BOLD:AAB9456 | 07-370 | ARC26899 | SCAFB982-07 | KY033900 |
| **ARGENTINIFORMES** |  |  |  |  |  |
| **Bathylagidae** |  |  |  |  |  |
| *Bathylagus euryops* | BOLD:AAB5134 | 07-383 | ARC26900 | SCAFB995-07 | KY033866 |
| *Bathylagus euryops* | BOLD:AAB5134 | 06-014 | ARC25677 | SCFAC351-06 | KY033867 |
| *Bathylagus euryops* | BOLD:AAB5134 | 09-352 | ARC28300 | SCFAD434-09 | KY033868 |
| *Bathylagus euryops* | BOLD:AAB5134 | 09-351 | ARC28300 | SCFAD433-09 | KY033869 |
| *Bathylagus euryops* | BOLD:AAB5134 | 06-1029 | ARC26375 | SCAFB213-07 | KY033870 |
| **ALEPOCEPHALIFORMES** |  |  |  |  |  |
| **Alepocephalidae** |  |  |  |  |  |
| *Xenodermichthys copei* | BOLD:AAB2780 | 07-377 | ARC26898 | SCAFB989-07 | KY033964 |
| *Xenodermichthys copei* | BOLD:AAB2780 | 06-389 | ARC25510 | SCFAC425-06 | KY033965 |
| *Xenodermichthys copei* | BOLD:AAB2780 | 06-373 | ARC25438 | SCFAC434-06 | KY033966 |
| **Platytroctidae** |  |  |  |  |  |
| *Holtbyrnia macrops** | BOLD:ABY9841 | 07-378 | ARC26883 | SCAFB990-07 | KY033902 |
| *Normichthys operosus* | BOLD:AAC3091 | 07-412 | ARC26889 | SCAFB1024-07 | KY033928 |
| *Normichthys operosus* | BOLD:AAC3091 | 07-413 | ARC26889 | SCAFB1025-07 | KY033929 |
| *Normichthys operosus* | BOLD:AAC3091 | 06-1026 | ARC26736 | SCAFB210-07 | KY033930 |
| **STOMIIFORMES** |  |  |  |  |  |
| **Gonostomatidae** |  |  |  |  |  |
| *Cyclothone microdon* | BOLD:AAB4944 | 07-409 | ARC26867 | SCAFB1021-07 | KY033897 |
| *Cyclothone microdon* | BOLD:AAB4944 | 07-368 | ARC26867 | SCAFB980-07 | KY033898 |
| *Cyclothone microdon* | BOLD:AAB4944 | 06-1039 | ARC26726 | SCAFB224-07 | KY033899 |
| *Gonostoma elongatum* | BOLD:AAB2394 | 06-592 | ARC26132 | SCFAC596-06 | KY033901 |
| **Sternoptychidae** |  |  |  |  |  |
| *Argyropelecus gigas* | BOLD:AAC0977 | 06-706 | ARC26156 | SCFAC787-06 | KY033865 |
| *Sternoptyx diaphana* | BOLD:AAI4421 | 07-388 | ARC26859 | SCAFB1000-07 | KY033949 |
| **Phosichthyidae** |  |  |  |  |  |
| *Polymetme corythaeola** | BOLD:AAA9250 | 07-213 | ARC26819 | SCAFB825-07 | KY033938 |
| **Stomiidae** |  |  |  |  |  |
| *Borostomias antarcticus* | BOLD:AAB5140 | 07-372 | ARC26909 | SCAFB984-07 | KY033872 |
| *Borostomias antarcticus* | BOLD:AAB5140 | 07-408 | ARC26909 | SCAFB1020-07 | KY033873 |
| *Borostomias mononema** | BOLD:AAF0984 | 07-381 | ARC26882 | SCAFB993-07 | KY033874 |
| *Chauliodus sloani* | BOLD:AAB1178 | 96-010 | ARC25838 | SCFAC671-06 | KY033880 |
| *Chauliodus sloani* | BOLD:AAB1178 | 07-361 | ARC26885 | SCAFB973-07 | KY033882 |
| *Chauliodus sloani* | BOLD:AAB1178 | 96-003 | ARC25835 | SCFAC667-06 | KY033883 |
| *Chauliodus sloani* | BOLD:AAB1179 | 07-216 | ARC26816 | SCAFB828-07 | KY033881 |
| *Malacosteus niger* | BOLD:AAB5515 | 07-396 | ARC26872 | SCAFB1008-07 | KY033909 |
| *Malacosteus niger* | BOLD:AAB5515 | 06-523 | ARC26079 | SCFAC575-06 | KY033910 |
| *Melanostomias bartonbeani** | BOLD:AAD8053 | 07-401 | ARC26887 | SCAFB1013-07 | KY033917 |
| *Stomias boa** | BOLD:AAB1180 | 06-821 | ARC26853 | SCFAC824-06 | KY033950 |
| *Stomias boa* | BOLD:AAB1180 | 06-033 | ARC25686 | SCFAC303-06 | KY033951 |
| *Stomias boa* | BOLD:AAB1180 | 96-006 | ARC25849 | SCFAC668-06 | KY033952 |
| *Stomias boa* | BOLD:AAB1180 | 06-1081 | ARC26619 | SCAFB268-07 | KY033953 |
| *Stomias boa* | BOLD:AAB1180 | 06-707 | ARC26152 | SCAFB526-07 | KY033954 |
| *Stomias boa* | BOLD:AAB1180 | 06-708 | ARC26142 | SCAFB527-07 | KY033955 |
| *Stomias boa* | BOLD:AAB1180 | 06-591 | ARC26158 | SCAFB497-07 | KY033956 |
| *Stomias boa* | BOLD:AAB1180 | 07-215 | ARC26812 | SCAFB827-07 | KY033957 |
| *Stomias boa* | BOLD:AAB1180 | 07-221 | ARC26820 | SCAFB833-07 | KY033958 |
| *Stomias boa* | BOLD:AAB1180 | 07-222 | ARC26820 | SCAFB834-07 | KY033959 |
| **AULOPIFORMES** |  |  |  |  |  |
| **Chlorophthalmidae** |  |  |  |  |  |
| *Chlorophthalmus agassizi* | BOLD:AAB2600 | 06-257 | ARC26002 | SCFAC469-06 | KY033887 |
| *Chlorophthalmus agassizi* | BOLD:AAB2600 | 05-953 | ARC25556 | SCFAC298-06 | KY033888 |
| *Chlorophthalmus agassizi* | BOLD:AAB2600 | 05-842 | ARC24884 | SCAFB094-07 | KY033889 |
| *Chlorophthalmus agassizi* | BOLD:AAB2600 | 06-386 | ARC25445 | SCFAC447-06 | KY033890 |
| *Chlorophthalmus agassizi* | BOLD:AAB2600 | 06-336 | ARC25767 | SCAFB360-07 | KY033891 |
| **Notosudidae** |  |  |  |  |  |
| *Scopelosaurus lepidus* | BOLD:AAC3164 | 07-391 | ARC26876 | SCAFB1003-07 | KY033943 |
| **Paralepididae** |  |  |  |  |  |
| *Arctozenus risso* | BOLD:AAA8780 | 06-275 | ARC25793 | SCFAC556-06 | KY033850 |
| *Arctozenus risso* | BOLD:AAA8780 | 06-918 | ARC26745 | SCFAC843-06 | KY033851 |
| *Arctozenus risso* | BOLD:AAA8780 | 07-226 | ARC26824 | SCAFB838-07 | KY033852 |
| *Arctozenus risso* | BOLD:AAA8780 | 05-785 | ARC25983 | SCFAC528-06 | KY033853 |
| *Arctozenus risso* | BOLD:AAA8780 | 06-702 | ARC26161 | SCFAC637-06 | KY033854 |
| *Arctozenus risso* | BOLD:AAA8780 | 06-778 | ARC26234 | SCAFB547-07 | KY033855 |
| *Arctozenus risso* | BOLD:AAA8780 | 06-521 | ARC26074 | SCAFB457-07 | KY033856 |
| *Arctozenus risso* | BOLD:AAA8780 | 06-520 | ARC26074 | SCFAC766-06 | KY033857 |
| *Arctozenus risso* | BOLD:AAA8780 | 07-365 | ARC26901 | SCAFB977-07 | KY033858 |
| *Arctozenus risso* | BOLD:AAA8780 | 06-586 | ARC26159 | SCAFB494-07 | KY033859 |
| *Arctozenus risso* | BOLD:AAA8780 | 06-527 | ARC26054 | SCAFB458-07 | KY033860 |
| *Arctozenus risso* | BOLD:AAA8780 | 07-225 | ARC26824 | SCAFB837-07 | KY033861 |
| *Arctozenus risso* | BOLD:AAA8780 | 06-526 | ARC26054 | SCFAC578-06 | KY033862 |
| *Arctozenus risso* | BOLD:AAA8780 | 06-525 | ARC26054 | SCFAC577-06 | KY033863 |
| *Arctozenus risso* | BOLD:AAA8780 | 06-1073 | ARC26724 | SCAFB259-07 | KY033864 |
| *Magnisudis atlantica* | BOLD:AAB9276 | 06-524 | ARC26046 | SCFAC576-06 | KY033908 |
| **MYCTOPHIFORMES** |  |  |  |  |  |
| **Myctophidae** |  |  |  |  |  |
| *Ceratoscopelus maderensis* | BOLD:AAC2875 | 07-212 | ARC26817 | SCAFB824-07 | KY033879 |
| *Lampadena speculigera* | BOLD:AAC2331 | 07-373 | ARC26914 | SCAFB985-07 | KY033906 |
| *Lampadena speculigera* | BOLD:AAC2331 | 07-411 | ARC26914 | SCAFB1023-07 | KY033907 |
| *Myctophum punctatum* | BOLD:AAB5848 | 07-369 | ARC26871 | SCAFB981-07 | KY033918 |
| *Nannobrachium atrum* | BOLD:AAB3779 | 96-011 | ARC25839 | SCFAC672-06 | KY033919 |
| *Notoscopelus elongatus* | BOLD:AAB7073 | 06-568 | ARC26077 | SCAFB484-07 | KY033931 |
| *Notoscopelus elongatus* | BOLD:AAB7073 | 06-567 | ARC26077 | SCFAC590-06 | KY033932 |
| *Notoscopelus elongatus* | BOLD:AAB7073 | 07-230 | ARC26815 | SCAFB842-07 | KY033933 |
| *Notoscopelus elongatus* | BOLD:AAB7073 | 07-371 | ARC26877 | SCAFB983-07 | KY033934 |
| **GADIFORMES** |  |  |  |  |  |
| **Macrouridae** |  |  |  |  |  |
| *Coryphaenoides guentheri** | BOLD:AAB9333 | 07-392 | ARC26905 | SCAFB1004-07 | KY033892 |
| *Coryphaenoides rupestris** | BOLD:AAE0666 | 06-010 | ARC25621 | SCAFB187-07 | KY033893 |
| *Coryphaenoides rupestris* | BOLD:AAE0666 | 09-384 | ARC28327 | SCFAD466-09 | KY033894 |
| *Coryphaenoides rupestris* | BOLD:AAE0666 | 06-009 | ARC25621 | SCAFB186-07 | KY033895 |
| *Nezumia bairdii* | BOLD:AAB9334 | 05-797 | ARC26099 | SCFAC530-06 | KY033921 |
| *Nezumia bairdii* | BOLD:AAB9334 | 06-277 | ARC25780 | SCAFB325-07 | KY033922 |
| *Nezumia bairdii* | BOLD:AAB9334 | 06-024 | ARC25687 | SCAFB189-07 | KY033923 |
| *Nezumia bairdii* | BOLD:AAB9334 | 06-675 | ARC26196 | SCAFB518-07 | KY033924 |
| *Nezumia bairdii* | BOLD:AAB9334 | 06-242 | ARC26004 | SCAFB307-07 | KY033925 |
| *Nezumia bairdii* | BOLD:AAB9334 | 06-064 | ARC25930 | SCAFB200-07 | KY033926 |
| *Nezumia bairdii* | BOLD:AAB9334 | 06-531 | ARC26056 | SCFAC579-06 | KY033927 |
| **LOPHIIFORMES** |  |  |  |  |  |
| **Ceratiidae** |  |  |  |  |  |
| *Ceratias holboelli* | BOLD:AAC1510 | 06-466 | ARC25607 | SCFAC572-06 | KY033876 |
| *Ceratias holboelli* | BOLD:AAC1510 | 07-347 | ARC26918 | SCAFB959-07 | KY033877 |
| *Ceratias holboelli* | BOLD:AAC1510 | 09-364 | ARC28308 | SCFAD446-09 | KY033878 |
| *Cryptopsaras couesii* | BOLD:AAC1509 | 96-002 | ARC25827 | SCFAC889-06 | KY033896 |
| **BERYCIFORMES** |  |  |  |  |  |
| **Melamphaidae** |  |  |  |  |  |
| *Poromitra megalops* | BOLD:AAC7683 | 07-414 | ARC26902 | SCAFB1026-07 | KY033939 |
| *Scopelogadus mizolepis* | BOLD:AAB8806 | 06-057 | ARC25884a | SCFAC341-06 | KY033940 |
| *Scopelogadus mizolepis* | BOLD:AAB8806 | 06-058 | ARC25884c | SCFAC353-06 | KY033941 |
| *Scopelogadus mizolepis* | BOLD:AAB8806 | 06-537 | ARC26076 | SCAFB465-07 | KY033942 |
| **TRACHICHTHYIFORMES** |  |  |  |  |  |
| **Anoplogastridae** |  |  |  |  |  |
| *Anoplogaster cornuta* | BOLD:AAB6136 | 07-349 | ARC26903 | SCAFB961-07 | KY033848 |
| *Anoplogaster cornuta* | BOLD:AAB6136 | 06-059 | ARC25879 | SCFAC365-06 | KY033849 |
| **SCORPAENIFORMES** |  |  |  |  |  |
| **Zoarcidae** |  |  |  |  |  |
| *Melanostigma atlanticum* | BOLD:AAC0509 | 05-786 | ARC25802 | SCFAC359-06 | KY033911 |
| *Melanostigma atlanticum* | BOLD:AAC0509 | 06-766 | ARC26266 | SCFAC798-06 | KY033912 |
| *Melanostigma atlanticum* | BOLD:AAC0509 | 06-723 | ARC26176 | SCFAC647-06 | KY033913 |
| *Melanostigma atlanticum* | BOLD:AAC0509 | 06-701 | ARC26209 | SCFAC636-06 | KY033914 |
| *Melanostigma atlanticum* | BOLD:AAC0509 | 06-307 | ARC25723 | SCFAC724-06 | KY033915 |
| *Melanostigma atlanticum* | BOLD:AAC0509 | 06-878 | ARC26280 | SCAFB575-07 | KY033916 |
| **Liparidae** |  |  |  |  |  |
| *Paraliparis bathybius** | BOLD:ABZ4374 | 06-1075 | ARC26711 | SCAFB261-07 | KY033935 |
| *Paraliparis calidus** | BOLD:AAG5288 | 06-815 | ARC26214 | SCFAC822-06 | KY033936 |
| *Paraliparis copei* | BOLD:ABZ4378 | 06-772 | ARC26229 | SCFAC803-06 | KY033937 |
| **PERCIFORMES** |  |  |  |  |  |
| **Howellidae** |  |  |  |  |  |
| *Howella sherborni** | BOLD:AAB5845 | 06-377 | ARC25441 (fish B) | SCFAC567-06 | KY033905 |
| *Howella sherborni* | BOLD:AAB5845 | 06-380 | ARC25441 (fish E) | SCFAC568-06 | KY033905 |
| *Howella sherborni* | BOLD:AAB5845 | 06-378 | ARC25441 (fish C) | SCFAC748-06 | KY033905 |
| **Caristiidae** |  |  |  |  |  |
| *Caristius fasciatus** | BOLD:AAD6436 | 96-012 | ARC25830 | SCFAC666-06 | KY033875 |
| **SCOMBRIFORMES** |  |  |  |  |  |
| **Trichiuridae** |  |  |  |  |  |
| *Benthodesmus tenuis** | BOLD:AAD2423 | 06-309 | ARC25730 | SCFAC725-06 | KY033871 |
| **TRACHINIFORMES** |  |  |  |  |  |
| **Chiasmodontidae** |  |  |  |  |  |
| *Chiasmodon niger* | BOLD:AAB4943 | 06-036 | ARC25684 | SCFAC337-06 | KY033884 |
| *Chiasmodon niger* | BOLD:AAB4943 | 06-611 | ARC26136 | SCFAC600-06 | KY033885 |
| *Chiasmodon niger* | BOLD:AAB4943 | 07-386 | ARC26865 | SCAFB998-07 | KY033886 |

*Species not found in ACMB
