# Supplementary material for: Barcoding Atlantic Canada’s mesopelagic and upper bathypelagic marine fishes
Source: PLoS One. 2017 Sep 20;12(9):e0185173. doi: 10.1371/journal.pone.0185173 (PMC5607201; doi:10.1371/journal.pone.0185173)
Supplement: S2 Table — (DOCX) [file pone.0185173.s004.docx]

**S2 Table. Mean and Maximum Intra-specific K2P Genetic Distances and Nearest Neighbour Inter-Specific Distance for each Species in the Atlantic Canada Mesopelagic and Upper Bathypelagic Marine Fishes Reference Data Set (BOLD Projects ACMB and ACMF).**

| **Order** | **Family** | **Species** | **Mean Intra-specific Distance** | **Maximum Intra-specific Distance** | **Nearest Neighbour Species** | **Distance to Nearest Neighbour** |
| --- | --- | --- | --- | --- | --- | --- |
| Anguilliformes | Synaphobranchidae | *Simenchelys parasitica* | 1.03 | 1.09 | *Synaphobranchus kaupii* | 17.42 |
| Anguilliformes | Synaphobranchidae | *Synaphobranchus kaupii* | 0.05 | 0.18 | *Simenchelys parasitica* | 17.42 |
| Anguilliformes | Congridae | *Xenomystax congroides* | N/A | N/A | *Synaphobranchus kaupii* | 18.72 |
| Anguilliformes | Derichthyidae | *Derichthys serpentinus* | 0.1 | 0.15 | *Nessorhamphus ingolfianus* | 19.82 |
| Anguilliformes | Derichthyidae | *Nessorhamphus ingolfianus* | N/A | N/A | *Poromitra crassiceps* | 19.24 |
| Anguilliformes | Nemichthyidae | *Avocettina infans* | N/A | N/A | *Serrivomer lanceolatoides* | 19.98 |
| Anguilliformes | Nemichthyidae | *Nemichthys scolopaceus* | 0 | 0 | *Serrivomer beanii* | 21.32 |
| Anguilliformes | Serrivomeridae | *Serrivomer beanii* | 0.11 | 0.17 | *Serrivomer lanceolatoides* | 11.43 |
| Anguilliformes | Serrivomeridae | *Serrivomer lanceolatoides* | 0.34 | 0.77 | *Serrivomer beanii* | 11.43 |
| Anguilliformes | Eurypharyngidae | *Eurypharynx pelecanoides* | 0.77 | 0.77 | *Xenomystax congroides* | 19.32 |
| Argentiniformes | Bathylagidae | *Bathylagichthys greyae* | 0.78 | 1.09 | *Bathylagus euryops* | 15.47 |
| Argentiniformes | Bathylagidae | *Bathylagus euryops* | 0.18 | 0.46 | *Dolicholagus longirostris* | 15.28 |
| Argentiniformes | Bathylagidae | *Dolicholagus longirostris* | 0.34 | 0.34 | *Bathylagus euryops* | 15.28 |
| Alepocephaliformes | Alepocephalidae | *Alepocephalus agassizii* | N/A | N/A | *Holtbyrnia macrops* | 14.47 |
| Alepocephaliformes | Alepocephalidae | *Xenodermichthys copei* | 0.45 | 0.93 | *Alepocephalus agassizii* | 17.57 |
| Alepocephaliformes | Platytroctidae | *Barbantus curvifrons* | N/A | N/A | *Maulisia microlepis* | 12.95 |
| Alepocephaliformes | Platytroctidae | *Holtbyrnia anomala* | 0 | 0 | *Holtbyrnia macrops* | 3.64 |
| Alepocephaliformes | Platytroctidae | *Holtbyrnia macrops* | N/A | N/A | *Holtbyrnia anomala* | 3.64 |
| Alepocephaliformes | Platytroctidae | *Maulisia microlepis* | N/A | N/A | *Normichthys operosus* | 5.81 |
| Alepocephaliformes | Platytroctidae | *Normichthys operosus* | 0.62 | 0.93 | *Maulisia microlepis* | 5.81 |
| Stomiiformes | Gonostomatidae | *Cyclothone microdon* | 0.10 | 0.31 | *Cyclothone pallida* | 20.81 |
| Stomiiformes | Gonostomatidae | *Cyclothone pallida* | 0 | 0 | *Cyclothone microdon* | 20.81 |
| Stomiiformes | Gonostomatidae | *Gonostoma atlanticum* | 0 | 0 | *Gonostoma elongatum* | 19.87 |
| Stomiiformes | Gonostomatidae | *Gonostoma elongatum* | 0.15 | 0.31 | *Sigmops bathyphilus* | 18.09 |
| Stomiiformes | Gonostomatidae | *Sigmops bathyphilus* | 0.19 | 0.19 | *Gonostoma elongatum* | 18.09 |
| Stomiiformes | Sternoptychidae | *Argyropelecus aculeatus* | 0.31 | 0.31 | *Argyropelecus gigas* | 16.80 |
| Stomiiformes | Sternoptychidae | *Argyropelecus gigas* | 0.18 | 0.46 | *Argyropelecus aculeatus* | 16.80 |
| Stomiiformes | Sternoptychidae | *Argyropelecus hemigymnus* | 0 | 0 | *Argyropelecus aculeatus* | 18.08 |
| Stomiiformes | Sternoptychidae | *Maurolicus weitzmani* | 0 | 0 | *Lobianchia dofleini* | 18.30 |
| Stomiiformes | Sternoptychidae | *Polyipnus clarus* | 0.11 | 0.17 | *Stomias boa* | 17.39 |
| Stomiiformes | Sternoptychidae | *Sternoptyx diaphana* | 0.31 | 0.31 | *Borostomias antarcticus* | 25.84 |
| Stomiiformes | Sternoptychidae | *Valenciennellus tripunctulatus* | N/A | N/A | *Polymetme corythaeola* | 19.37 |
| Stomiiformes | Phosichthyidae | *Polymetme corythaeola* | N/A | N/A | *Borostomias antarcticus* | 17.58 |
| Stomiiformes | Phosichthyidae | *Vinciguerria nimbaria* | 0 | 0 | *Scopeloberyx opisthopterus* | 21.58 |
| Stomiiformes | Stomiidae | *Bathophilus vaillanti* | N/A | N/A | *Alepisaurus ferox* | 22.89 |
| Stomiiformes | Stomiidae | *Borostomias antarcticus* | 1.09 | 1.88 | *Borostomias mononema* | 12.72 |
| Stomiiformes | Stomiidae | *Borostomias mononema* | N/A | N/A | *Borostomias antarcticus* | 12.72 |
| Stomiiformes | Stomiidae | *Chauliodus sloani* | 5.36 | 13.14 | *Bathylagichthys greyae* | 20.17 |
| Stomiiformes | Stomiidae | *Flagellostomias boureei* | 0.15 | 0.15 | *Leptostomias gladiator* | 13.42 |
| Stomiiformes | Stomiidae | *Idiacanthus fasciola* | N/A | N/A | *Borostomias antarcticus* | 21.06 |
| Stomiiformes | Stomiidae | *Leptostomias gladiator* | N/A | N/A | *Flagellostomias boureei* | 13.42 |
| Stomiiformes | Stomiidae | *Malacosteus niger* | 0.13 | 0.31 | *Pachystomias microdon* | 16.85 |
| Stomiiformes | Stomiidae | *Melanostomias bartonbeani* | N/A | N/A | *Leptostomias gladiator* | 18.60 |
| Stomiiformes | Stomiidae | *Pachystomias microdon* | 0 | 0 | *Malacosteus niger* | 16.85 |
| Stomiiformes | Stomiidae | *Photostomias guernei* | 0.50 | 0.80 | *Bathylagichthys greyae* | 23.40 |
| Stomiiformes | Stomiidae | *Stomias boa* | 0.55 | 1.24 | *Borostomias antarcticus* | 14.98 |
| Aulopiformes | Chlorophthalmidae | *Chlorophthalmus agassizi* | 0.44 | 0.84 | *Arctozenus risso* | 18.48 |
| Aulopiformes | Notosudidae | *Scopelosaurus lepidus* | 6.16 | 9.25 | *Omosudis lowii* | 17.89 |
| Aulopiformes | Giganturidae | *Gigantura chuni* | N/A | N/A | *Alepisaurus ferox* | 21.19 |
| Aulopiformes | Paralepididae | *Arctozenus risso* | 0.59 | 1.29 | *Chlorophthalmus agassizi* | 18.48 |
| Aulopiformes | Paralepididae | *Magnisudis atlantica* | 0.41 | 0.62 | *Omosudis lowii* | 17.74 |
| Aulopiformes | Evermannellidae | *Evermannella balbo* | 0.32 | 0.62 | *Omosudis lowii* | 19.62 |
| Aulopiformes | Alepisauridae | *Alepisaurus brevirostris* | N/A | N/A | *Alepisaurus ferox* | 0.46 |
| Aulopiformes | Alepisauridae | *Alepisaurus ferox* | 3.30 | 6.51 | *Alepisaurus brevirostris* | 0.46 |
| Aulopiformes | Alepisauridae | *Omosudis lowii* | 0.72 | 0.93 | *Alepisaurus ferox* | 17.38 |
| Myctophiformes | Myctophidae | *Benthosema glaciale* | 0.83 | 0.83 | *Protomyctophum arcticum* | 14.77 |
| Myctophiformes | Myctophidae | *Bolinichthys indicus* | N/A | N/A | *Bolinichthys photothorax* | 8.24 |
| Myctophiformes | Myctophidae | *Bolinichthys photothorax* | N/A | N/A | *Bolinichthys indicus* | 8.24 |
| Myctophiformes | Myctophidae | *Ceratoscopelus maderensis* | 0.55 | 1.09 | *Ceratoscopelus warmingii* | 8.88 |
| Myctophiformes | Myctophidae | *Ceratoscopelus warmingii* | N/A | N/A | *Ceratoscopelus maderensis* | 8.88 |
| Myctophiformes | Myctophidae | *Diaphus dumerilii* | N/A | N/A | *Diaphus mollis* | 13.84 |
| Myctophiformes | Myctophidae | *Diaphus mollis* | N/A | N/A | *Diaphus dumerilii* | 13.84 |
| Myctophiformes | Myctophidae | *Hygophum hygomii* | 0.21 | 0.31 | *Lobianchia dofleini* | 16.93 |
| Myctophiformes | Myctophidae | *Lampadena speculigera* | 0.13 | 0.38 | *Diaphus dumerilii* | 15.70 |
| Myctophiformes | Myctophidae | *Lampanyctus festivus* | N/A | N/A | *Nannobrachium atrum* | 10.25 |
| Myctophiformes | Myctophidae | *Lampanyctus macdonaldi* | 0.21 | 0.31 | *Nannobrachium atrum* | 9.11 |
| Myctophiformes | Myctophidae | *Lampanyctus photonotus* | N/A | N/A | *Nannobrachium atrum* | 11.78 |
| Myctophiformes | Myctophidae | *Lampanyctus pusillus* | N/A | N/A | *Lampanyctus festivus* | 12.93 |
| Myctophiformes | Myctophidae | *Lepidophanes guentheri* | 0 | 0 | *Ceratoscopelus warmingii* | 14.88 |
| Myctophiformes | Myctophidae | *Lobianchia dofleini* | 0.23 | 0.46 | *Diaphus dumerilii* | 16.00 |
| Myctophiformes | Myctophidae | *Myctophum affine* | 0 | 0 | *Myctophum punctatum* | 18.52 |
| Myctophiformes | Myctophidae | *Myctophum punctatum* | 0.26 | 0.72 | *Symbolophorus veranyi* | 16.71 |
| Myctophiformes | Myctophidae | *Nannobrachium atrum* | 0.51 | 0.91 | *Lampanyctus macdonaldi* | 9.11 |
| Myctophiformes | Myctophidae | *Nannobrachium lineatum* | N/A | N/A | *Nannobrachium atrum* | 11.23 |
| Myctophiformes | Myctophidae | *Notoscopelus bolini* | 0.23 | 0.46 | *Notoscopelus elongatus* | 6.25 |
| Myctophiformes | Myctophidae | *Notoscopelus elongatus* | 0.15 | 0.47 | *Notoscopelus bolini* | 6.25 |
| Myctophiformes | Myctophidae | *Notoscopelus resplendens* | 1.47 | 2.32 | *Notoscopelus bolini* | 8.98 |
| Myctophiformes | Myctophidae | *Protomyctophum arcticum* | 0 | 0 | *Benthosema glaciale* | 14.77 |
| Myctophiformes | Myctophidae | *Symbolophorus veranyi* | 0.40 | 0.77 | *Protomyctophum arcticum* | 16.30 |
| Myctophiformes | Myctophidae | *Taaningichthys bathyphilus* | 0.31 | 0.46 | *Lampadena speculigera* | 16.66 |
| Gadiformes | Macrouridae | *Coryphaenoides guentheri* | N/A | N/A | *Coryphaenoides rupestris* | 12.97 |
| Gadiformes | Macrouridae | *Coryphaenoides rupestris* | 0.10 | 0.16 | *Coryphaenoides guentheri* | 12.97 |
| Gadiformes | Macrouridae | *Nezumia bairdii* | 0.08 | 0.31 | *Laemonema barbatulum* | 19.73 |
| Gadiformes | Moridae | *Laemonema barbatulum* | 0.50 | 0.69 | *Notoscopelus resplendens* | 19.63 |
| Gadiformes | Melanonidae | *Melanonus zugmayeri* | N/A | N/A | *Laemonema barbatulum* | 20.34 |
| Ophidiiformes | Ophidiidae | *Lamprogrammus brunswigi* | N/A | N/A | *Melanonus zugmayeri* | 20.61 |
| Lophiiformes | Melanocetidae | *Melanocetus johnsonii* | N/A | N/A | *Chaenophryne longiceps* | 16.41 |
| Lophiiformes | Oneirodidae | *Chaenophryne longiceps* | N/A | N/A | *Lophodolos acanthognathus* | 13.45 |
| Lophiiformes | Oneirodidae | *Lophodolos acanthognathus* | 0 | 0 | *Oneirodes bradburyae* | 11.81 |
| Lophiiformes | Oneirodidae | *Oneirodes bradburyae* | N/A | N/A | *Lophodolos acanthognathus* | 11.81 |
| Lophiiformes | Ceratiidae | *Ceratias holboelli* | 0.15 | 0.31 | *Melanocetus johnsonii* | 18.18 |
| Lophiiformes | Ceratiidae | *Cryptopsaras couesii* | 0.31 | 0.47 | *Ceratias holboelli* | 21.25 |
| Lophiiformes | Linophrynidae | *Haplophryne mollis* | 0 | 0 | *Argyropelecus gigas* | 23.22 |
| Beryciformes | Melamphaidae | *Melamphaes suborbitalis* | 0.78 | 1.29 | *Scopeloberyx opisthopterus* | 14.76 |
| Beryciformes | Melamphaidae | *Poromitra capito* | N/A | N/A | *Poromitra crassiceps* | 8.26 |
| Beryciformes | Melamphaidae | *Poromitra crassiceps* | 0.81 | 0.81 | *Poromitra capito* | 8.26 |
| Beryciformes | Melamphaidae | *Poromitra megalops* | 0.54 | 0.77 | *Poromitra capito* | 13.99 |
| Beryciformes | Melamphaidae | *Scopeloberyx opisthopterus* | 0.62 | 0.77 | *Poromitra crassiceps* | 13.24 |
| Beryciformes | Melamphaidae | *Scopelogadus beanii* | N/A | N/A | *Scopelogadus mizolepis* | 0.78 |
| Beryciformes | Melamphaidae | *Scopelogadus mizolepis* | 0.08 | 0.17 | *Scopelogadus beanii* | 0.78 |
| Beryciformes | Rondeletiidae | *Rondeletia loricata* | 0.72 | 1.03 | *Anoplogaster cornuta* | 16.59 |
| Beryciformes | Cetomimidae | *Cetostoma regani* | 0 | 0 | *Rondeletia loricata* | 18.14 |
| Trachichthyiformes | Anoplogastridae | *Anoplogaster cornuta* | 0.62 | 1.36 | *Rondeletia loricata* | 16.56 |
| Zeiformes | Grammicolepididae | *Xenolepidichthys dalgleishi* | N/A | N/A | *Lampanyctus pusillus* | 19.32 |
| Scorpaeniformes | Zoarcidae | *Melanostigma atlanticum* | 0.36 | 0.77 | *Howella sherborni* | 18.73 |
| Scorpaeniformes | Liparidae | *Paraliparis bathybius* | N/A | N/A | *Paraliparis calidus* | 6.92 |
| Scorpaeniformes | Liparidae | *Paraliparis calidus* | N/A | N/A | *Paraliparis bathybius* | 6.92 |
| Scorpaeniformes | Liparidae | *Paraliparis copei* | 0.23 | 0.32 | *Paraliparis bathybius* | 8.36 |
| Perciformes | Howellidae | *Howella brodiei* | N/A | N/A | *Anoplogaster cornuta* | 17.6 |
| Perciformes | Howellidae | *Howella sherborni* | 0 | 0 | *Melanostigma atlanticum* | 18.73 |
| Perciformes | Caristiidae | *Caristius fasciatus* | N/A | N/A | *Chiasmodon niger* | 19.8 |
| Perciformes | Priacanthidae | *Priacanthus arenatus* | N/A | N/A | *Myctophum affine* | 22.22 |
| Perciformes | Chaetodontidae | *Chaetodon ocellatus* | N/A | N/A | *Leptostomias gladiator* | 21.02 |
| Scombriformes | Gempylidae | *Diplospinus multistriatus* | N/A | N/A | *Caristius fasciatus* | 20.01 |
| Scombriformes | Gempylidae | *Nealotus tripes* | 1.08 | 1.72 | *Bathylagichthys greyae* | 18.34 |
| Scombriformes | Trichiuridae | *Benthodesmus tenuis* | N/A | N/A | *Nealotus tripes* | 19.13 |
| Trachiniformes | Chiasmodontidae | *Chiasmodon niger* | 0.68 | 1.4 | *Caristius fasciatus* | 19.8 |
| Trachiniformes | Chiasmodontidae | *Pseudoscopelus astronesthidens* | N/A | N/A | *Nealotus tripes* | 19.58 |

N/A: Species represented in reference data set by a single sequence.
