# Supplementary material for: Barcoding Atlantic Canada’s mesopelagic and upper bathypelagic marine fishes
Source: PLoS One. 2017 Sep 20;12(9):e0185173. doi: 10.1371/journal.pone.0185173 (PMC5607201; doi:10.1371/journal.pone.0185173)
Supplement: S3 Table — (DOCX) [file pone.0185173.s005.docx]

**S3 Table. K2P Genetic Distances Between Individuals for each Intra-generic, Inter-specific Pair of Sequences in the Atlantic Canada Mesopelagic and Upper Bathypelagic Marine Fishes Reference Data Set (BOLD Projects ACMB and ACMF).**

| **First Individual of Pair** | | **Second Individual of Pair** | |  |
| --- | --- | --- | --- | --- |
| **BOLD Process ID** | **Species** | **BOLD Process ID** | **Species** | **Genetic Distance** |
| **ANGUILLIFORMES** |  |  |  |  |
| **Serrivomeridae** |  |  |  |  |
| SCFAC887-06 | *Serrivomer beanii* | SCAFB1151-09 | *Serrivomer lanceolatoides* | 11.71 |
| SCFAC887-06 | *Serrivomer beanii* | SCAFB1143-09 | *Serrivomer lanceolatoides* | 11.52 |
| SCFAC887-06 | *Serrivomer beanii* | SCAFB1207-09 | *Serrivomer lanceolatoides* | 11.90 |
| SCFAC887-06 | *Serrivomer beanii* | SCAFB1113-09 | *Serrivomer lanceolatoides* | 11.52 |
| SCFAC887-06 | *Serrivomer beanii* | SCAFB1292-09 | *Serrivomer lanceolatoides* | 11.70 |
| SCFAC669-06 | *Serrivomer beanii* | SCAFB1151-09 | *Serrivomer lanceolatoides* | 12.32 |
| SCFAC669-06 | *Serrivomer beanii* | SCAFB1143-09 | *Serrivomer lanceolatoides* | 12.04 |
| SCFAC669-06 | *Serrivomer beanii* | SCAFB1207-09 | *Serrivomer lanceolatoides* | 12.46 |
| SCFAC669-06 | *Serrivomer beanii* | SCAFB1113-09 | *Serrivomer lanceolatoides* | 12.04 |
| SCFAC669-06 | *Serrivomer beanii* | SCAFB1292-09 | *Serrivomer lanceolatoides* | 12.23 |
| SCFAC315-06 | *Serrivomer beanii* | SCAFB1151-09 | *Serrivomer lanceolatoides* | 11.71 |
| SCFAC315-06 | *Serrivomer beanii* | SCAFB1143-09 | *Serrivomer lanceolatoides* | 11.46 |
| SCFAC315-06 | *Serrivomer beanii* | SCAFB1207-09 | *Serrivomer lanceolatoides* | 11.84 |
| SCFAC315-06 | *Serrivomer beanii* | SCAFB1113-09 | *Serrivomer lanceolatoides* | 11.46 |
| SCFAC315-06 | *Serrivomer beanii* | SCAFB1292-09 | *Serrivomer lanceolatoides* | 11.64 |
| SCAFB1218-09 | *Serrivomer beanii* | SCAFB1151-09 | *Serrivomer lanceolatoides* | 11.70 |
| SCAFB1218-09 | *Serrivomer beanii* | SCAFB1143-09 | *Serrivomer lanceolatoides* | 11.45 |
| SCAFB1218-09 | *Serrivomer beanii* | SCAFB1207-09 | *Serrivomer lanceolatoides* | 11.83 |
| SCAFB1218-09 | *Serrivomer beanii* | SCAFB1113-09 | *Serrivomer lanceolatoides* | 11.45 |
| SCAFB1218-09 | *Serrivomer beanii* | SCAFB1292-09 | *Serrivomer lanceolatoides* | 11.62 |
| **ALEPOCEPHALIFORMES** |  |  |  |  |
| **Platytroctidae** |  |  |  |  |
| SCAFB990-07 | *Holtbyrnia macrops* | SCAFB1154-09 | *Holtbyrnia anomala* | 3.64 |
| SCAFB990-07 | *Holtbyrnia macrops* | SCAFB1258-09 | *Holtbyrnia anomala* | 3.64 |
| **STOMIIFORMES** |  |  |  |  |
| **Gonostomatidae** |  |  |  |  |
| SCAFB1021-07 | *Cyclothone microdon* | SCAFB1145-09 | *Cyclothone pallida* | 20.81 |
| SCAFB1021-07 | *Cyclothone microdon* | SCAFB1115-09 | *Cyclothone pallida* | 21.32 |
| SCAFB980-07 | *Cyclothone microdon* | SCAFB1145-09 | *Cyclothone pallida* | 21.20 |
| SCAFB980-07 | *Cyclothone microdon* | SCAFB1115-09 | *Cyclothone pallida* | 21.32 |
| SCAFB224-07 | *Cyclothone microdon* | SCAFB1145-09 | *Cyclothone pallida* | 20.81 |
| SCAFB224-07 | *Cyclothone microdon* | SCAFB1115-09 | *Cyclothone pallida* | 21.32 |
| SCAFB1281-09 | *Cyclothone microdon* | SCAFB1145-09 | *Cyclothone pallida* | 20.81 |
| SCFAD050-09 | *Cyclothone microdon* | SCAFB1145-09 | *Cyclothone pallida* | 20.81 |
| SCAFB1299-09 | *Cyclothone microdon* | SCAFB1145-09 | *Cyclothone pallida* | 20.81 |
| SCAFB1281-09 | *Cyclothone microdon* | SCAFB1115-09 | *Cyclothone pallida* | 21.32 |
| SCFAD050-09 | *Cyclothone microdon* | SCAFB1115-09 | *Cyclothone pallida* | 21.32 |
| SCAFB1299-09 | *Cyclothone microdon* | SCAFB1115-09 | *Cyclothone pallida* | 21.32 |
| SCAFB1189-09 | *Gonostoma elongatum* | SCAFB1079-09 | *Gonostoma atlanticum* | 20.09 |
| SCAFB1189-09 | *Gonostoma elongatum* | SCAFB1277-09 | *Gonostoma atlanticum* | 20.19 |
| SCAFB1229-09 | *Gonostoma elongatum* | SCAFB1079-09 | *Gonostoma atlanticum* | 20.30 |
| SCAFB1229-09 | *Gonostoma elongatum* | SCAFB1277-09 | *Gonostoma atlanticum* | 20.52 |
| SCAFB1197-09 | *Gonostoma elongatum* | SCAFB1079-09 | *Gonostoma atlanticum* | 19.98 |
| SCAFB1197-09 | *Gonostoma elongatum* | SCAFB1277-09 | *Gonostoma atlanticum* | 20.19 |
| SCFAC596-06 | *Gonostoma elongatum* | SCAFB1079-09 | *Gonostoma atlanticum* | 19.87 |
| SCFAC596-06 | *Gonostoma elongatum* | SCAFB1277-09 | *Gonostoma atlanticum* | 20.08 |
| **Sternoptychidae** |  |  |  |  |
| SCAFB1125-09 | *Argyropelecus gigas* | SCAFB1232-09 | *Argyropelecus aculeatus* | 17.21 |
| SCAFB1125-09 | *Argyropelecus gigas* | SCAFB1055-09 | *Argyropelecus aculeatus* | 16.91 |
| SCAFB1117-09 | *Argyropelecus gigas* | SCAFB1232-09 | *Argyropelecus aculeatus* | 17.21 |
| SCAFB1117-09 | *Argyropelecus gigas* | SCAFB1055-09 | *Argyropelecus aculeatus* | 17.00 |
| SCAFB1233-09 | *Argyropelecus gigas* | SCAFB1232-09 | *Argyropelecus aculeatus* | 16.91 |
| SCAFB1233-09 | *Argyropelecus gigas* | SCAFB1055-09 | *Argyropelecus aculeatus* | 16.80 |
| SCAFB1116-09 | *Argyropelecus gigas* | SCAFB1232-09 | *Argyropelecus aculeatus* | 17.21 |
| SCAFB1116-09 | *Argyropelecus gigas* | SCAFB1055-09 | *Argyropelecus aculeatus* | 17.00 |
| SCFAC787-06 | *Argyropelecus gigas* | SCAFB1232-09 | *Argyropelecus aculeatus* | 17.51 |
| SCFAC787-06 | *Argyropelecus gigas* | SCAFB1055-09 | *Argyropelecus aculeatus* | 17.40 |
| SCAFB1125-09 | *Argyropelecus gigas* | SCFAD005-09 | *Argyropelecus hemigymnus* | 23.44 |
| SCAFB1125-09 | *Argyropelecus gigas* | SCAFB1216-09 | *Argyropelecus hemigymnus* | 23.58 |
| SCAFB1117-09 | *Argyropelecus gigas* | SCFAD005-09 | *Argyropelecus hemigymnus* | 23.44 |
| SCAFB1117-09 | *Argyropelecus gigas* | SCAFB1216-09 | *Argyropelecus hemigymnus* | 23.58 |
| SCAFB1233-09 | *Argyropelecus gigas* | SCFAD005-09 | *Argyropelecus hemigymnus* | 23.21 |
| SCAFB1233-09 | *Argyropelecus gigas* | SCAFB1216-09 | *Argyropelecus hemigymnus* | 23.34 |
| SCAFB1116-09 | *Argyropelecus gigas* | SCFAD005-09 | *Argyropelecus hemigymnus* | 23.44 |
| SCAFB1116-09 | *Argyropelecus gigas* | SCAFB1216-09 | *Argyropelecus hemigymnus* | 23.58 |
| SCFAC787-06 | *Argyropelecus gigas* | SCFAD005-09 | *Argyropelecus hemigymnus* | 23.90 |
| SCFAC787-06 | *Argyropelecus gigas* | SCAFB1216-09 | *Argyropelecus hemigymnus* | 24.02 |
| SCFAD005-09 | *Argyropelecus hemigymnus* | SCAFB1232-09 | *Argyropelecus aculeatus* | 18.69 |
| SCFAD005-09 | *Argyropelecus hemigymnus* | SCAFB1055-09 | *Argyropelecus aculeatus* | 18.25 |
| SCAFB1216-09 | *Argyropelecus hemigymnus* | SCAFB1232-09 | *Argyropelecus aculeatus* | 18.51 |
| SCAFB1216-09 | *Argyropelecus hemigymnus* | SCAFB1055-09 | *Argyropelecus aculeatus* | 18.08 |
| **Stomiidae** |  |  |  |  |
| SCAFB993-07 | *Borostomias mononema* | SCAFB1176-09 | *Borostomias antarcticus* | 13.58 |
| SCAFB993-07 | *Borostomias mononema* | SCAFB1084-09 | *Borostomias antarcticus* | 13.76 |
| SCAFB993-07 | *Borostomias mononema* | SCAFB1263-09 | *Borostomias antarcticus* | 13.71 |
| SCAFB993-07 | *Borostomias mononema* | SCAFB984-07 | *Borostomias antarcticus* | 12.72 |
| SCAFB993-07 | *Borostomias mononema* | SCAFB1020-07 | *Borostomias antarcticus* | 13.31 |
| **AULOPIFORMES** |  |  |  |  |
| **Alepisauridae** |  |  |  |  |
| SCAFB1278-09 | *Alepisaurus ferox* | SCAFB1127-09 | *Alepisaurus brevirostris* | 0.46 |
| SCAFB1169-09 | *Alepisaurus ferox* | SCAFB1127-09 | *Alepisaurus brevirostris* | 0.46 |
| SCAFB1030-09 | *Alepisaurus ferox* | SCAFB1127-09 | *Alepisaurus brevirostris* | 6.17 |
| SCAFB1118-09 | *Alepisaurus ferox* | SCAFB1127-09 | *Alepisaurus brevirostris* | 0.46 |
| **MYCTOPHIFORMES** |  |  |  |  |
| **Myctophidae** |  |  |  |  |
| SCAFB1224-09 | *Bolinichthys photothorax* | SCAFB1234-09 | *Bolinichthys indicus* | 8.24 |
| SCAFB1314-09 | *Ceratoscopelus warmingii* | SCAFB824-07 | *Ceratoscopelus maderensis* | 10.06 |
| SCAFB1314-09 | *Ceratoscopelus warmingii* | SCAFB1062-09 | *Ceratoscopelus maderensis* | 9.75 |
| SCAFB1314-09 | *Ceratoscopelus warmingii* | SCAFB1249-09 | *Ceratoscopelus maderensis* | 9.15 |
| SCAFB1314-09 | *Ceratoscopelus warmingii* | SCAFB1063-09 | *Ceratoscopelus maderensis* | 9.56 |
| SCAFB1314-09 | *Ceratoscopelus warmingii* | SCAFB1214-09 | *Ceratoscopelus maderensis* | 9.33 |
| SCAFB1314-09 | *Ceratoscopelus warmingii* | SCAFB1144-09 | *Ceratoscopelus maderensis* | 8.88 |
| SCAFB1293-09 | *Diaphus mollis* | SCAFB1225-09 | *Diaphus dumerilii* | 13.84 |
| SCAFB1312-09 | *Lampanyctus festivus* | SCAFB1092-09 | *Lampanyctus macdonaldi* | 11.33 |
| SCAFB1312-09 | *Lampanyctus festivus* | SCAFB1222-09 | *Lampanyctus macdonaldi* | 11.65 |
| SCAFB1312-09 | *Lampanyctus festivus* | SCAFB1091-09 | *Lampanyctus macdonaldi* | 11.46 |
| SCAFB1312-09 | *Lampanyctus festivus* | SCAFB1241-09 | *Lampanyctus photonotus* | 12.50 |
| SCAFB1312-09 | *Lampanyctus festivus* | SCAFB1276-09 | *Lampanyctus pusillus* | 12.93 |
| SCAFB1092-09 | *Lampanyctus macdonaldi* | SCAFB1241-09 | *Lampanyctus photonotus* | 12.74 |
| SCAFB1222-09 | *Lampanyctus macdonaldi* | SCAFB1241-09 | *Lampanyctus photonotus* | 12.68 |
| SCAFB1091-09 | *Lampanyctus macdonaldi* | SCAFB1241-09 | *Lampanyctus photonotus* | 12.48 |
| SCAFB1092-09 | *Lampanyctus macdonaldi* | SCAFB1276-09 | *Lampanyctus pusillus* | 13.48 |
| SCAFB1222-09 | *Lampanyctus macdonaldi* | SCAFB1276-09 | *Lampanyctus pusillus* | 13.41 |
| SCAFB1091-09 | *Lampanyctus macdonaldi* | SCAFB1276-09 | *Lampanyctus pusillus* | 13.60 |
| SCAFB1276-09 | *Lampanyctus pusillus* | SCAFB1241-09 | *Lampanyctus photonotus* | 15.72 |
| SCFAD039-09 | *Myctophum punctatum* | SCAFB1193-09 | *Myctophum affine* | 19.08 |
| SCFAD039-09 | *Myctophum punctatum* | SCAFB1290-09 | *Myctophum affine* | 18.90 |
| SCFAD039-09 | *Myctophum punctatum* | SCAFB1208-09 | *Myctophum affine* | 18.90 |
| SCFAD063-09 | *Myctophum punctatum* | SCAFB1193-09 | *Myctophum affine* | 19.08 |
| SCFAD063-09 | *Myctophum punctatum* | SCAFB1290-09 | *Myctophum affine* | 18.80 |
| SCFAD063-09 | *Myctophum punctatum* | SCAFB1208-09 | *Myctophum affine* | 18.80 |
| SCAFB1210-09 | *Myctophum punctatum* | SCAFB1193-09 | *Myctophum affine* | 18.81 |
| SCAFB1210-09 | *Myctophum punctatum* | SCAFB1290-09 | *Myctophum affine* | 18.71 |
| SCAFB1210-09 | *Myctophum punctatum* | SCAFB1208-09 | *Myctophum affine* | 18.71 |
| SCAFB1301-09 | *Myctophum punctatum* | SCAFB1193-09 | *Myctophum affine* | 18.81 |
| SCAFB1301-09 | *Myctophum punctatum* | SCAFB1290-09 | *Myctophum affine* | 18.71 |
| SCAFB1301-09 | *Myctophum punctatum* | SCAFB1208-09 | *Myctophum affine* | 18.71 |
| SCAFB1141-09 | *Myctophum punctatum* | SCAFB1193-09 | *Myctophum affine* | 19.48 |
| SCAFB1141-09 | *Myctophum punctatum* | SCAFB1290-09 | *Myctophum affine* | 19.21 |
| SCAFB1141-09 | *Myctophum punctatum* | SCAFB1208-09 | *Myctophum affine* | 19.21 |
| SCFAD040-09 | *Myctophum punctatum* | SCAFB1193-09 | *Myctophum affine* | 19.08 |
| SCFAD040-09 | *Myctophum punctatum* | SCAFB1290-09 | *Myctophum affine* | 18.88 |
| SCFAD040-09 | *Myctophum punctatum* | SCAFB1208-09 | *Myctophum affine* | 18.88 |
| SCAFB1142-09 | *Myctophum punctatum* | SCAFB1193-09 | *Myctophum affine* | 18.57 |
| SCAFB1142-09 | *Myctophum punctatum* | SCAFB1290-09 | *Myctophum affine* | 18.52 |
| SCAFB1142-09 | *Myctophum punctatum* | SCAFB1208-09 | *Myctophum affine* | 18.52 |
| SCAFB981-07 | *Myctophum punctatum* | SCAFB1193-09 | *Myctophum affine* | 19.08 |
| SCAFB981-07 | *Myctophum punctatum* | SCAFB1290-09 | *Myctophum affine* | 18.71 |
| SCAFB981-07 | *Myctophum punctatum* | SCAFB1208-09 | *Myctophum affine* | 18.71 |
| SCAFB1090-09 | *Nannobrachium lineatum* | SCFAC672-06 | *Nannobrachium atrum* | 11.23 |
| SCAFB1090-09 | *Nannobrachium lineatum* | SCAFB1089-09 | *Nannobrachium atrum* | 11.61 |
| SCAFB1090-09 | *Nannobrachium lineatum* | SCAFB1205-09 | *Nannobrachium atrum* | 11.42 |
| SCAFB1090-09 | *Nannobrachium lineatum* | SCAFB1093-09 | *Nannobrachium atrum* | 11.92 |
| SCAFB1090-09 | *Nannobrachium lineatum* | SCAFB1094-09 | *Nannobrachium atrum* | 11.42 |
| SCAFB1124-09 | *Notoscopelus elongatus* | SCAFB1060-09 | *Notoscopelus bolini* | 6.85 |
| SCAFB1124-09 | *Notoscopelus elongatus* | SCAFB1202-09 | *Notoscopelus bolini* | 6.67 |
| SCAFB1124-09 | *Notoscopelus elongatus* | SCAFB1095-09 | *Notoscopelus bolini* | 6.64 |
| SCAFB1124-09 | *Notoscopelus elongatus* | SCAFB1061-09 | *Notoscopelus bolini* | 6.64 |
| SCFAD067-09 | *Notoscopelus elongatus* | SCAFB1060-09 | *Notoscopelus bolini* | 6.64 |
| SCFAD067-09 | *Notoscopelus elongatus* | SCAFB1202-09 | *Notoscopelus bolini* | 6.47 |
| SCFAD067-09 | *Notoscopelus elongatus* | SCAFB1095-09 | *Notoscopelus bolini* | 6.50 |
| SCFAD067-09 | *Notoscopelus elongatus* | SCAFB1061-09 | *Notoscopelus bolini* | 6.50 |
| SCFAD068-09 | *Notoscopelus elongatus* | SCAFB1060-09 | *Notoscopelus bolini* | 6.74 |
| SCFAD068-09 | *Notoscopelus elongatus* | SCAFB1202-09 | *Notoscopelus bolini* | 6.56 |
| SCFAD068-09 | *Notoscopelus elongatus* | SCAFB1095-09 | *Notoscopelus bolini* | 6.25 |
| SCFAD068-09 | *Notoscopelus elongatus* | SCAFB1061-09 | *Notoscopelus bolini* | 6.60 |
| SCAFB1240-09 | *Notoscopelus elongatus* | SCAFB1060-09 | *Notoscopelus bolini* | 6.67 |
| SCAFB1240-09 | *Notoscopelus elongatus* | SCAFB1202-09 | *Notoscopelus bolini* | 6.50 |
| SCAFB1240-09 | *Notoscopelus elongatus* | SCAFB1095-09 | *Notoscopelus bolini* | 6.53 |
| SCAFB1240-09 | *Notoscopelus elongatus* | SCAFB1061-09 | *Notoscopelus bolini* | 6.53 |
| SCAFB484-07 | *Notoscopelus elongatus* | SCAFB1060-09 | *Notoscopelus bolini* | 6.99 |
| SCAFB484-07 | *Notoscopelus elongatus* | SCAFB1202-09 | *Notoscopelus bolini* | 6.81 |
| SCAFB484-07 | *Notoscopelus elongatus* | SCAFB1095-09 | *Notoscopelus bolini* | 6.85 |
| SCFAC590-06 | *Notoscopelus elongatus* | SCAFB1060-09 | *Notoscopelus bolini* | 6.64 |
| SCFAC590-06 | *Notoscopelus elongatus* | SCAFB1202-09 | *Notoscopelus bolini* | 6.47 |
| SCFAC590-06 | *Notoscopelus elongatus* | SCAFB1095-09 | *Notoscopelus bolini* | 6.50 |
| SCFAC590-06 | *Notoscopelus elongatus* | SCAFB1061-09 | *Notoscopelus bolini* | 6.50 |
| SCAFB484-07 | *Notoscopelus elongatus* | SCAFB1061-09 | *Notoscopelus bolini* | 6.85 |
| SCAFB842-07 | *Notoscopelus elongatus* | SCAFB1060-09 | *Notoscopelus bolini* | 6.64 |
| SCAFB842-07 | *Notoscopelus elongatus* | SCAFB1202-09 | *Notoscopelus bolini* | 6.47 |
| SCAFB842-07 | *Notoscopelus elongatus* | SCAFB1095-09 | *Notoscopelus bolini* | 6.50 |
| SCAFB842-07 | *Notoscopelus elongatus* | SCAFB1061-09 | *Notoscopelus bolini* | 6.50 |
| SCAFB983-07 | *Notoscopelus elongatus* | SCAFB1060-09 | *Notoscopelus bolini* | 6.64 |
| SCAFB983-07 | *Notoscopelus elongatus* | SCAFB1202-09 | *Notoscopelus bolini* | 6.47 |
| SCAFB983-07 | *Notoscopelus elongatus* | SCAFB1095-09 | *Notoscopelus bolini* | 6.50 |
| SCAFB983-07 | *Notoscopelus elongatus* | SCAFB1061-09 | *Notoscopelus bolini* | 6.50 |
| SCAFB484-07 | *Notoscopelus elongatus* | SCAFB1035-09 | *Notoscopelus resplendens* | 11.79 |
| SCAFB484-07 | *Notoscopelus elongatus* | SCAFB1215-09 | *Notoscopelus resplendens* | 11.97 |
| SCAFB484-07 | *Notoscopelus elongatus* | SCAFB1201-09 | *Notoscopelus resplendens* | 12.92 |
| SCAFB484-07 | *Notoscopelus elongatus* | SCAFB1036-09 | *Notoscopelus resplendens* | 12.02 |
| SCFAC590-06 | *Notoscopelus elongatus* | SCAFB1035-09 | *Notoscopelus resplendens* | 11.58 |
| SCFAC590-06 | *Notoscopelus elongatus* | SCAFB1215-09 | *Notoscopelus resplendens* | 11.97 |
| SCFAC590-06 | *Notoscopelus elongatus* | SCAFB1201-09 | *Notoscopelus resplendens* | 12.92 |
| SCFAC590-06 | *Notoscopelus elongatus* | SCAFB1036-09 | *Notoscopelus resplendens* | 12.23 |
| SCAFB842-07 | *Notoscopelus elongatus* | SCAFB1035-09 | *Notoscopelus resplendens* | 11.58 |
| SCAFB842-07 | *Notoscopelus elongatus* | SCAFB1215-09 | *Notoscopelus resplendens* | 11.97 |
| SCAFB842-07 | *Notoscopelus elongatus* | SCAFB1201-09 | *Notoscopelus resplendens* | 12.92 |
| SCAFB842-07 | *Notoscopelus elongatus* | SCAFB1036-09 | *Notoscopelus resplendens* | 12.23 |
| SCAFB983-07 | *Notoscopelus elongatus* | SCAFB1035-09 | *Notoscopelus resplendens* | 11.58 |
| SCAFB983-07 | *Notoscopelus elongatus* | SCAFB1215-09 | *Notoscopelus resplendens* | 11.97 |
| SCAFB983-07 | *Notoscopelus elongatus* | SCAFB1201-09 | *Notoscopelus resplendens* | 12.92 |
| SCAFB983-07 | *Notoscopelus elongatus* | SCAFB1036-09 | *Notoscopelus resplendens* | 12.23 |
| SCAFB1124-09 | *Notoscopelus elongatus* | SCAFB1035-09 | *Notoscopelus resplendens* | 11.38 |
| SCFAD067-09 | *Notoscopelus elongatus* | SCAFB1035-09 | *Notoscopelus resplendens* | 11.58 |
| SCFAD068-09 | *Notoscopelus elongatus* | SCAFB1035-09 | *Notoscopelus resplendens* | 12.00 |
| SCAFB1240-09 | *Notoscopelus elongatus* | SCAFB1035-09 | *Notoscopelus resplendens* | 11.65 |
| SCAFB1124-09 | *Notoscopelus elongatus* | SCAFB1215-09 | *Notoscopelus resplendens* | 11.85 |
| SCFAD067-09 | *Notoscopelus elongatus* | SCAFB1215-09 | *Notoscopelus resplendens* | 11.97 |
| SCFAD068-09 | *Notoscopelus elongatus* | SCAFB1215-09 | *Notoscopelus resplendens* | 12.35 |
| SCAFB1240-09 | *Notoscopelus elongatus* | SCAFB1215-09 | *Notoscopelus resplendens* | 12.04 |
| SCAFB1124-09 | *Notoscopelus elongatus* | SCAFB1201-09 | *Notoscopelus resplendens* | 13.18 |
| SCFAD067-09 | *Notoscopelus elongatus* | SCAFB1201-09 | *Notoscopelus resplendens* | 12.92 |
| SCFAD068-09 | *Notoscopelus elongatus* | SCAFB1201-09 | *Notoscopelus resplendens* | 13.12 |
| SCAFB1240-09 | *Notoscopelus elongatus* | SCAFB1201-09 | *Notoscopelus resplendens* | 12.98 |
| SCAFB1124-09 | *Notoscopelus elongatus* | SCAFB1036-09 | *Notoscopelus resplendens* | 12.02 |
| SCFAD067-09 | *Notoscopelus elongatus* | SCAFB1036-09 | *Notoscopelus resplendens* | 12.23 |
| SCFAD068-09 | *Notoscopelus elongatus* | SCAFB1036-09 | *Notoscopelus resplendens* | 12.43 |
| SCAFB1240-09 | *Notoscopelus elongatus* | SCAFB1036-09 | *Notoscopelus resplendens* | 12.23 |
| SCAFB1035-09 | *Notoscopelus resplendens* | SCAFB1060-09 | *Notoscopelus bolini* | 9.18 |
| SCAFB1035-09 | *Notoscopelus resplendens* | SCAFB1202-09 | *Notoscopelus bolini* | 8.98 |
| SCAFB1035-09 | *Notoscopelus resplendens* | SCAFB1095-09 | *Notoscopelus bolini* | 9.38 |
| SCAFB1035-09 | *Notoscopelus resplendens* | SCAFB1061-09 | *Notoscopelus bolini* | 8.98 |
| SCAFB1215-09 | *Notoscopelus resplendens* | SCAFB1060-09 | *Notoscopelus bolini* | 9.61 |
| SCAFB1215-09 | *Notoscopelus resplendens* | SCAFB1202-09 | *Notoscopelus bolini* | 9.43 |
| SCAFB1215-09 | *Notoscopelus resplendens* | SCAFB1095-09 | *Notoscopelus bolini* | 9.84 |
| SCAFB1215-09 | *Notoscopelus resplendens* | SCAFB1061-09 | *Notoscopelus bolini* | 9.47 |
| SCAFB1201-09 | *Notoscopelus resplendens* | SCAFB1060-09 | *Notoscopelus bolini* | 9.42 |
| SCAFB1201-09 | *Notoscopelus resplendens* | SCAFB1202-09 | *Notoscopelus bolini* | 9.24 |
| SCAFB1201-09 | *Notoscopelus resplendens* | SCAFB1095-09 | *Notoscopelus bolini* | 9.65 |
| SCAFB1201-09 | *Notoscopelus resplendens* | SCAFB1061-09 | *Notoscopelus bolini* | 9.28 |
| SCAFB1036-09 | *Notoscopelus resplendens* | SCAFB1060-09 | *Notoscopelus bolini* | 9.69 |
| SCAFB1036-09 | *Notoscopelus resplendens* | SCAFB1202-09 | *Notoscopelus bolini* | 9.50 |
| SCAFB1036-09 | *Notoscopelus resplendens* | SCAFB1095-09 | *Notoscopelus bolini* | 9.88 |
| SCAFB1036-09 | *Notoscopelus resplendens* | SCAFB1061-09 | *Notoscopelus bolini* | 9.50 |
| **GADIFORMES** |  |  |  |  |
| **Macrouridae** |  |  |  |  |
| SCAFB187-07 | *Coryphaenoides rupestris* | SCAFB1004-07 | *Coryphaenoides guentheri* | 13.10 |
| SCFAD466-09 | *Coryphaenoides rupestris* | SCAFB1004-07 | *Coryphaenoides guentheri* | 13.32 |
| SCAFB186-07 | *Coryphaenoides rupestris* | SCAFB1004-07 | *Coryphaenoides guentheri* | 12.97 |
| **BERYCIFORMES** |  |  |  |  |
| **Melamphaidae** |  |  |  |  |
| SCAFB1192-09 | *Poromitra crassiceps* | SCAFB1245-09 | *Poromitra capito* | 8.74 |
| SCAFB1171-09 | *Poromitra crassiceps* | SCAFB1245-09 | *Poromitra capito* | 8.26 |
| SCAFB1282-09 | *Poromitra megalops* | SCAFB1192-09 | *Poromitra crassiceps* | 15.61 |
| SCAFB1282-09 | *Poromitra megalops* | SCAFB1171-09 | *Poromitra crassiceps* | 15.06 |
| SCAFB1195-09 | *Poromitra megalops* | SCAFB1192-09 | *Poromitra crassiceps* | 15.61 |
| SCAFB1195-09 | *Poromitra megalops* | SCAFB1171-09 | *Poromitra crassiceps* | 15.06 |
| SCAFB1238-09 | *Poromitra megalops* | SCAFB1192-09 | *Poromitra crassiceps* | 15.82 |
| SCAFB1238-09 | *Poromitra megalops* | SCAFB1171-09 | *Poromitra crassiceps* | 15.26 |
| SCAFB1026-07 | *Poromitra megalops* | SCAFB1192-09 | *Poromitra crassiceps* | 15.40 |
| SCAFB1026-07 | *Poromitra megalops* | SCAFB1171-09 | *Poromitra crassiceps* | 15.26 |
| SCAFB1282-09 | *Poromitra megalops* | SCAFB1245-09 | *Poromitra capito* | 14.19 |
| SCAFB1195-09 | *Poromitra megalops* | SCAFB1245-09 | *Poromitra capito* | 14.19 |
| SCAFB1238-09 | *Poromitra megalops* | SCAFB1245-09 | *Poromitra capito* | 13.99 |
| SCAFB1026-07 | *Poromitra megalops* | SCAFB1245-09 | *Poromitra capito* | 14.78 |
| SCAFB1044-09 | *Scopelogadus mizolepis* | SCAFB1043-09 | *Scopelogadus beanii* | 0.93 |
| SCFAC341-06 | *Scopelogadus mizolepis* | SCAFB1043-09 | *Scopelogadus beanii* | 0.84 |
| SCFAC353-06 | *Scopelogadus mizolepis* | SCAFB1043-09 | *Scopelogadus beanii* | 0.80 |
| SCAFB465-07 | *Scopelogadus mizolepis* | SCAFB1043-09 | *Scopelogadus beanii* | 0.78 |
| **SCORPAENIFORMES** |  |  |  |  |
| **Liparidae** |  |  |  |  |
| SCAFB261-07 | *Paraliparis bathybius* | SCAFB1137-09 | *Paraliparis copei* | 8.00 |
| SCAFB261-07 | *Paraliparis bathybius* | SCAFB1300-09 | *Paraliparis copei* | 8.24 |
| SCAFB261-07 | *Paraliparis bathybius* | SCAFB1136-09 | *Paraliparis copei* | 8.20 |
| SCAFB261-07 | *Paraliparis bathybius* | SCFAC803-06 | *Paraliparis copei* | 8.39 |
| SCFAC822-06 | *Paraliparis calidus* | SCAFB1137-09 | *Paraliparis copei* | 12.33 |
| SCFAC822-06 | *Paraliparis calidus* | SCAFB1300-09 | *Paraliparis copei* | 12.33 |
| SCFAC822-06 | *Paraliparis calidus* | SCAFB1136-09 | *Paraliparis copei* | 12.14 |
| SCFAC822-06 | *Paraliparis calidus* | SCFAC803-06 | *Paraliparis copei* | 12.06 |
| SCFAC822-06 | *Paraliparis calidus* | SCAFB261-07 | *Paraliparis bathybius* | 6.77 |
| **PERCIFORMES** |  |  |  |  |
| **Howellidae** |  |  |  |  |
| SCFAC567-06 | *Howella sherborni* | SCAFB1283-09 | *Howella brodiei* | 20.12 |
| SCFAC568-06 | *Howella sherborni* | SCAFB1283-09 | *Howella brodiei* | 20.12 |
| SCFAC748-06 | *Howella sherborni* | SCAFB1283-09 | *Howella brodiei* | 20.75 |
